# A critical review on diabetes mellitus type 1 and type 2 management approaches: from lifestyle modification to current and novel targets and therapeutic agents

**DOI:** 10.3389/fendo.2024.1440456

**Published:** 2024-10-18

**Authors:** Bantayehu Addis Tegegne, Adane Adugna, Aderaw Yenet, Wubetu Yihunie Belay, Yared Yibeltal, Abebe Dagne, Zigale Hibstu Teffera, Gashaw Azanaw Amare, Desalegn Abebaw, Haymanot Tewabe, Rahel Belete Abebe, Tirsit Ketsela Zeleke

**Affiliations:** ^1^ Department of Pharmacy, College of Medicine and Health Science, Debre Markos University, Debre Markos, Ethiopia; ^2^ Department of Medical Laboratory Science, College of Medicine and Health Science, Debre Markos University, Debre Markos, Ethiopia; ^3^ Department of Clinical Pharmacy, School of Pharmacy, College of Medicine and Health Science, University of Gondar, Gondar, Ethiopia

**Keywords:** diabetes mellitus, lifestyle modification, current anti-diabetics, novel drugs and targets, probiotics, phtomedicines

## Abstract

Diabetes mellitus (DM) has emerged as an international health epidemic due to its rapid rise in prevalence. Consequently, scientists and or researchers will continue to find novel, safe, effective, and affordable anti-diabetic medications. The goal of this review is to provide a thorough overview of the role that lifestyle changes play in managing diabetes, as well as the standard medications that are currently being used to treat the condition and the most recent advancements in the development of novel medical treatments that may be used as future interventions for the disease. A literature search was conducted using research databases such as PubMed, Web of Science, Scopus, ScienceDirect, Wiley Online Library, Google Scholar, etc. Data were then abstracted from these publications using words or Phrases like “pathophysiology of diabetes”, “Signe and symptoms of diabetes”, “types of diabetes”, “major risk factors and complication of diabetes”, “diagnosis of diabetes”, “lifestyle modification for diabetes”, “current antidiabetic agents”, and “novel drugs and targets for diabetes management” that were published in English and had a strong scientific foundation. Special emphasis was given to the importance of lifestyle modification, as well as current, novel, and emerging/promising drugs and targets helpful for the management of both T1DM and T2DM.

## 1 Introduction

### 1.1 Background

Diabetes mellitus (DM) has become a major worldwide healthcare concern due to its sharp increase in occurrence. DM affects people of all ages, socioeconomic backgrounds, and demographic subgroups in nearly every country on the planet (1). Although DM affects everyone on the earth, both its incidence and death rates are gradually rising. Its incidence is highest in Low to Middle Income Countries (LMICs) (2). Ancient peoples recognized illnesses that shared the characteristics of DM. Determining the causes of hyperglycemia through study was made possible by the realization that diabetes was not a single illness (3).

DM is now recognized as an illness that exhibits the “iceberg” phenomenon, meaning that the number of documented instances represents just a small portion of the enormous number of unidentified cases. It is a chronic illness stemming from either inadequate synthesis of insulin by the pancreas or inefficient utilization of that production by the body. Uncontrolled diabetes frequently results in hyperglycemia, or elevated blood glucose, which over time seriously damages numerous bodily systems, including the blood vessels and neurons (4, 5).

### 1.2 Signs and/or symptoms of diabetes mellitus

**Figure 1:**
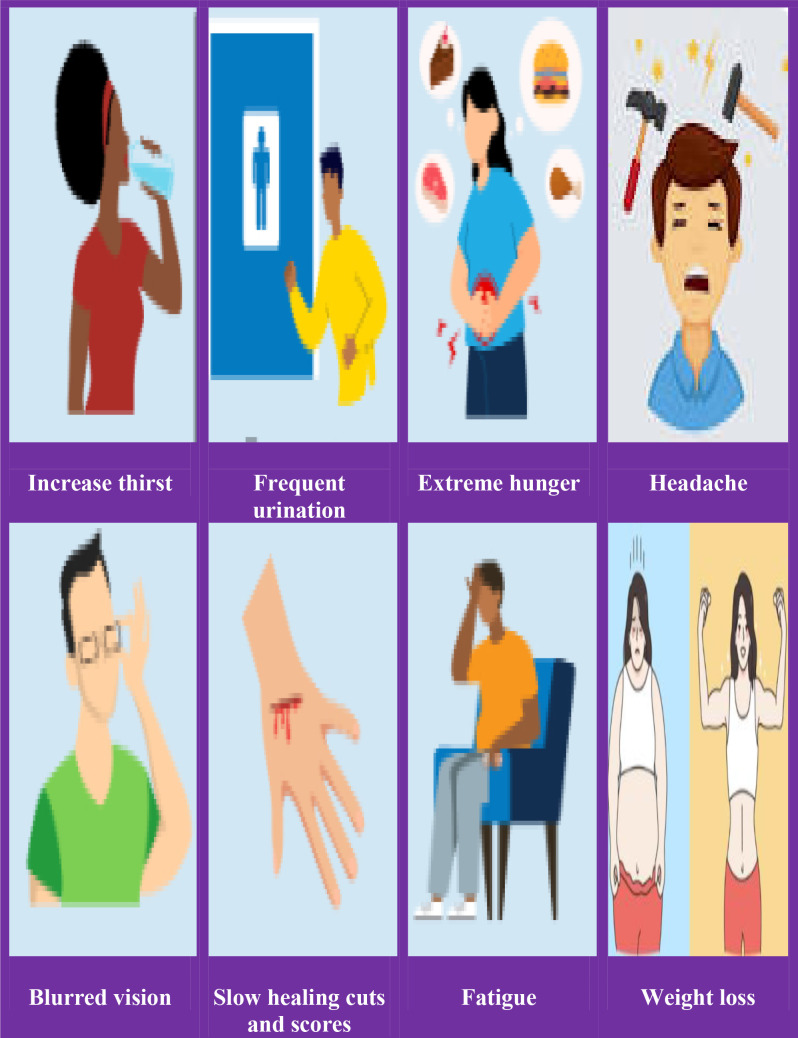
A schematic representation of the common signs and symptoms of hyperglycemia.

The following are some of the early signs and/or symptoms of hyperglycemia: increased thirst (polydipsia and/or hunger), frequent urination, headache, blurred vision, fatigue, weight loss, vaginal yeast infection, skin infection, slow healing of wounds and scores are symptoms of long-term hyperglycemia (**Figure 1**).

### 1.3 Etiologic classification of diabetes mellitus

**Figure 2:**
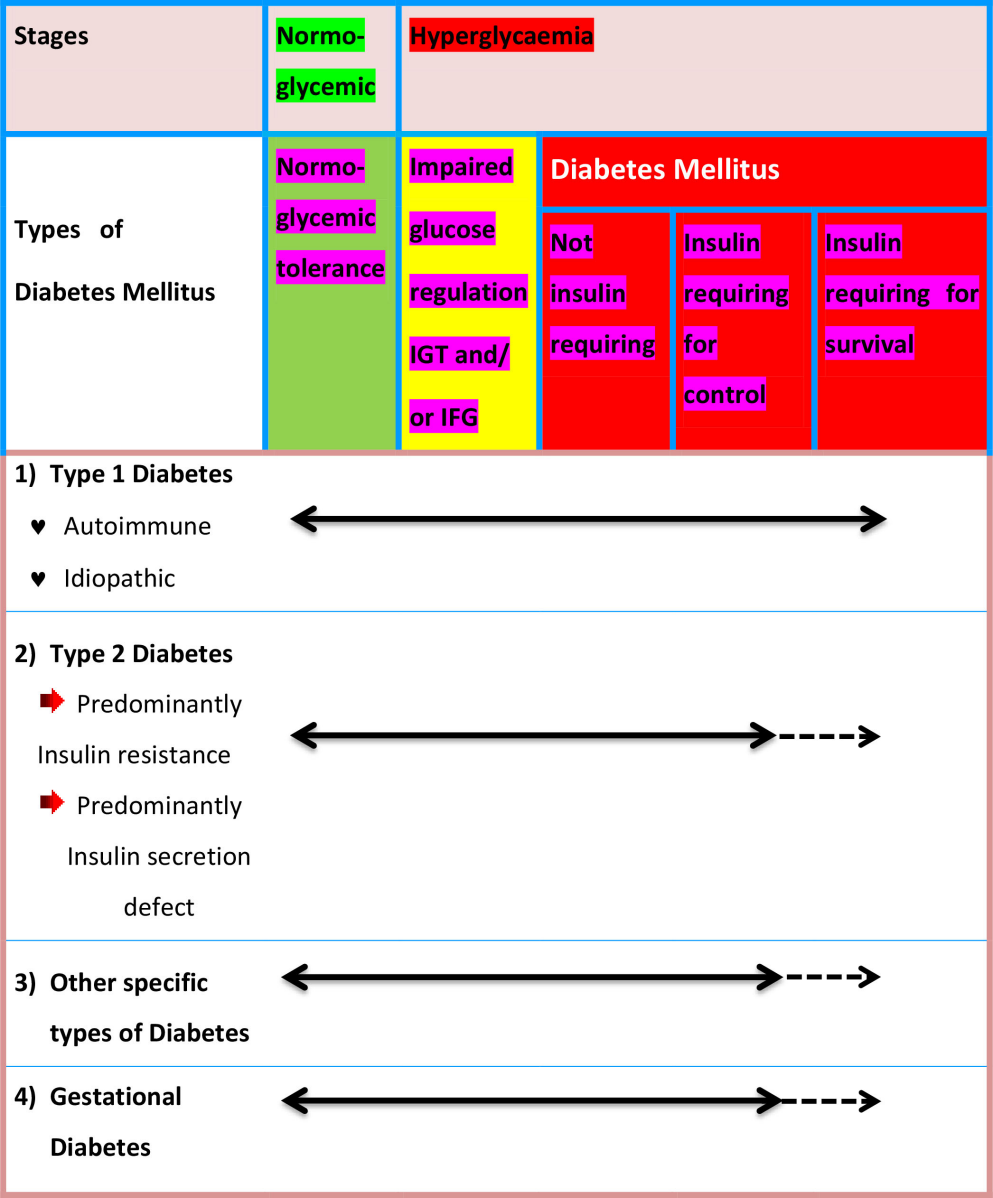
Disorders of glycaemia: etiological categories and clinical phases. IGT, Impaired Glucose tolerance; IFG, Impaired Fasting Glucose.

Many diabetics are challenging to place into a single group, and determining a person’s type of diabetes typically depends on the conditions that were in place at that point of the diagnosis. Nonetheless, four main classifications of diabetes are generally accepted throughout a wide range of literary works, specifically: 1) Diabetes mellitus type 1 (autoimmune pancreatic β-cell destruction) 2) Type 2 diabetes mellitus (constant loss of pancreatic β-cell insulin secretion often within the framework of insulin resistance); 3) gestational diabetes mellitus, which was previously unknown and discovered during the second or third trimester of pregnancy; 4) other specific forms of diabetes, which result from different causes, including drug- or chemical-induced diabetes and monogenic diabetes syndromes, which include neonatal-diabetes and maturity-onset diabetes of the Young (6, 7). The etiological categories and clinical phases of diabetes are shown in **Figure 2** (8).

### 1.4 Pathophysiology of diabetes mellitus

#### 1.4.1 Type 1 diabetes mellitus (T1DM)

**Figure 3:**
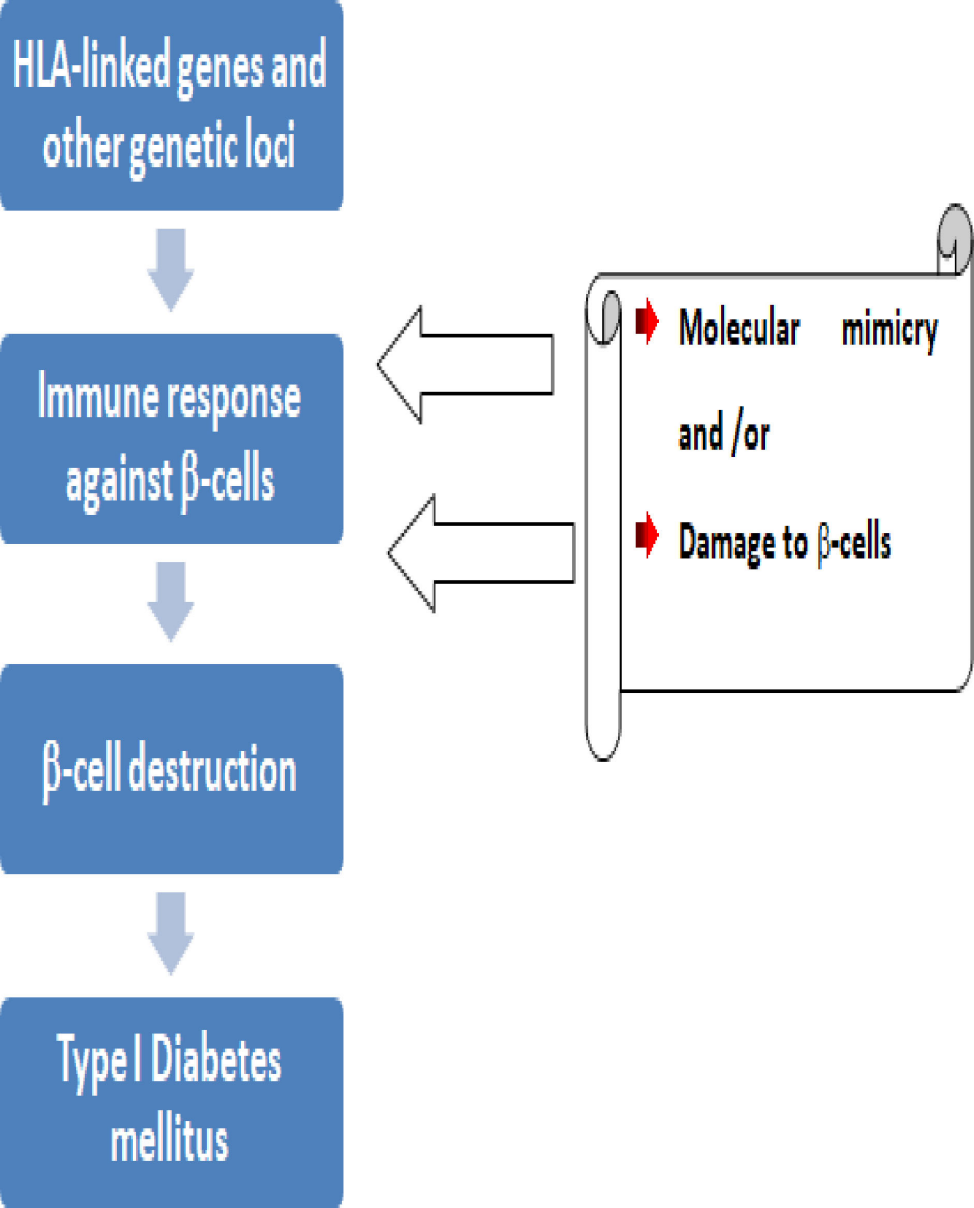
A diagrammatic representation of the pathogenesis of T1DM.

T1DM is a complicated disorder that results from both genetic risk (**Figure 3**) and environmental triggers that alter immune pathways. T1DM arises from the cell-mediated autoimmune destruction of insulin producing pancreatic β-cells by CD4^+^ and CD8^+^ T-cells and macrophages. There are four different markers for this pancreatic β-cell destruction namely; 1) islet cell autoantibodies, 2) autoantibodies to insulin, 3) autoantibodies to glutamic acid decarboxylase (GAD65), and 4) autoantibodies to the tyrosine phosphatases IA-2 and IA-2b (9, 10).

The majority of T1DM patients also had detectable anti-insulin antibodies before starting insulin therapy, with about 85% of them having circulating islet cell antibodies (11). 57 genetic risk loci for T1DM have been found by genome-wide association studies. Most of the islet cell antibodies are directed against Glutamic Acid Decarboxylase (GAD), an enzyme that produces a specific neurotransmitter called γ-amino butyric acid within pancreatic β-cells. GAD can trigger the immune system to secret autoantibodies against healthy cells. GAD antibodies are one of a group of diabetes-associated autoantibodies that order the immune system to destroy pancreatic β-cells. These ultimately lead to a deficiency of insulin secretion which results in the metabolic disturbance associated with T1DM (12).

The pancreas has played an important role in managing macronutrient digestion as well as metabolism (energy balance by secreting multiple enzymes for digestion and the pancreas hormones) (13). Specifically, pancreatic β-cells, which secrete the hormone insulin, are the basic adjudicator of glucose homeostasis (14). When there is a loss of insulin secretion, the function of pancreatic α-cells is also abnormal and there is excessive secretion of glucagon in T1DM patients. The patients have hyperglycemia due to abnormal glucagon release from pancreatic α-cells. The hyperglycemic action of glucagon is mediated by an increase in hepatic glycogenolysis and gluconeogenesis, which boosts endogenous glucose production. Furthermore, glucagon is the primary human hormone that promotes insulin resistance, particularly in the liver (15).

Insulin deficiency minimizes several gene expressions necessary for target tissues to respond normally to insulin-like glucokinase in the liver and the glucose transporter-4 (GLUT_4_) in adipose tissue; uncontrolled lipolysis and elevated free fatty acids (FFAs) levels in the plasma, which suppresses glucose metabolism in peripheral tissues like skeletal muscle. About 10% of insulin-stimulated glucose absorption via the GLUT_4_ receptors is shown by adipocytes, compared to 60–70% in skeletal muscle (16–18).

#### 1.4.2 Type 2 diabetes mellitus (T2DM)

T2DM is by far the most common DM that accounts for >90% of cases affecting people of all age groups (19). The two main frequently cited hallmarks of T2DM are impaired insulin secretion due to dysfunction of the pancreatic β-cell and impaired insulin action due to insulin resistance. The mass of pancreatic β-cells transforms capable of elevating insulin supply and compensating for excessive and abnormal demand in situations where insulin resistance predominates (20, 21). Generally, the mode of inheritance for T2DM is unclear, except for the Maturity-Onset Diabetes of the Young (MODY). MODY is inherited as an autosomal dominant trait resulting from mutations in the glucokinase gene on chromosome 7p (22).

### 1.5 Diabetes mellitus primary risk factors

#### 1.5.1 NADH and reductive stress

The majority of the electrons produced during the aerobic metabolism of glucose are stored in NADH for the formation of adenosine triphosphate and oxygen reduction. Consequently, Reductive Stress (RS) might result from an excess of NADH, which is a reducing molecule. RS is not the reverse of oxidative stress, but it leads to oxidative stress. Therefore, RS followed by oxidative stress comprises the main mechanism of hyperglycaemia-induced metabolic syndrome (23, 24).

**Figure 4:**
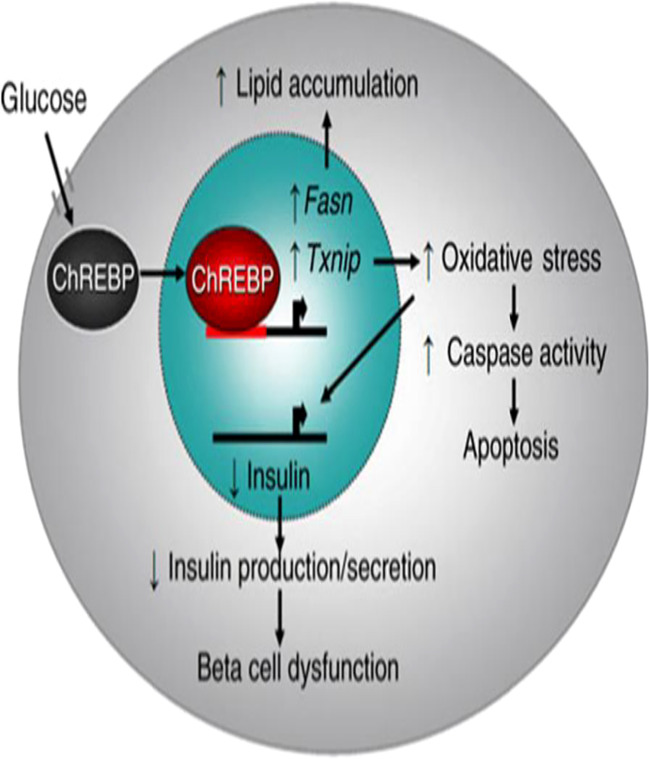
Model of carbohydrate response element-binding protein-mediated β-Cell glucotoxicity: Adapted From (25).

Excessive generation of reactive oxygen species (ROS) and NADH is brought on by prolonged hyperglycemia. This inhibits glyceraldehyde-3-phosphate dehydrogenase (GAPDH) activity resulting formation of excessive ROS that further exacerbates oxidative stress. When complex-I in the mitochondria is overloaded with NADH, the mitochondrial electron transport chain produces more ROS. Additionally, excessive NADH can also inhibit the glycolytic pathway, the pyruvate dehydrogenase complex, and the Krebs cycle, which in turn leads to more passage of glucose via the polyol pathway. As a result, glucose is reduced by the enzyme aldose reductase to form sorbitol, and the formed sorbitol is then converted to fructose by another enzyme sorbitol dehydrogenase. In this pathway, NADPH is converted to NADH in two-step reactions that lead to a redox mismatch between NAD^+^ and NADH. As the ratio of NAD^+^/NADH decreases, RS can ensue. Moreover, the pancreas produces carbohydrate response element-binding protein (ChREBP) which is a transcription factor up-regulating target genes fatty acid synthase (Fasn) and Thioredoxin-interacting protein (Txnip), leading to lipid accumulation, increased oxidative stress, stimulated caspase activity, apoptosis, down-regulate transcription of the insulin gene, and impaired insulin secretion (**Figure 4**) (25–29).

#### 1.5.2 Oxidative stress (OS)

OS plays a crucial role in the development of diabetes. More than half of diseases have resulted in secondary to excessive production of free radicals (30). OS condition arose owing to the high level of NADH, achieving the transition from RS to OS. The oxidation of cellular machinery is caused by free radicals, which are reactive entities that are constantly present in the human body.

**Figure 5:**
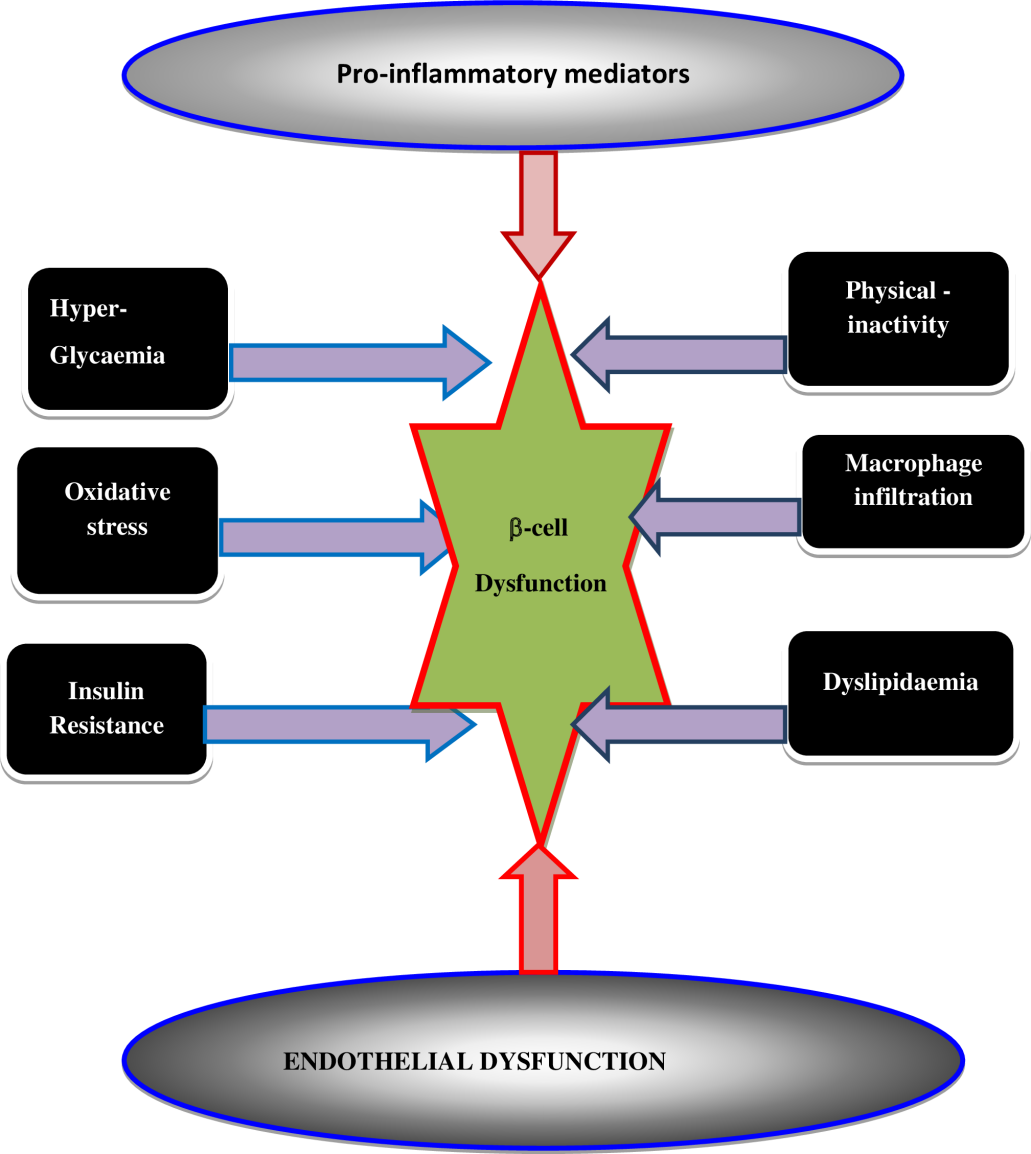
A diagrammatic illustration of the mechanism of β-cell dysfunction.

High level of nutrients (glucose and or fatty acid) in the blood leads to the generation of ROS. An increase in the levels of ROS ultimately leads to increased OS in a variety of tissues. Any imbalance between ROS and antioxidants leads to the production of a condition known as OS that leads to DM incidence. Intracellular stress-associated pathways are triggered when a cell and/or tissue is overtaken by OS (30–32). OS may itself potentiate the generation of ROS along with other pro-inflammatory cytokines and chemokines around the β-cells that disrupt the blood flow into the β-cells and abolish its function. β-cells dysfunction is induced by multiple risk factors (**Figure 5**) including; 1) optimal glucolipotoxicity (hyperglycemia and dyslipidemia), which can impact the progress of insulin resistance, OS, and/or endothelial cell dysfunction, 2) the activation of pro-inflammatory mediators and macrophage infiltration thereby may provoke the raid of T2DM as summarized in **Figure 5** (33).

#### 1.5.3 Genetics and insulin resistance

Genetics is among the major risk factors in the case of T2DM patients who have inherited genes from parents that enable their tissues resistant to insulin (34). Aside from that, stress response pathways inside cells are induced and inter-organ communication networks mediated by certain peptide hormones and cytokines cause insulin resistance (IR). Likewise, contribute to the risk of T2DM. IR is associated with flaws in the uptake and oxidation of glucose, low glycogen synthesis, and a fall in the capacity to suppress lipid oxidation to a lesser extent. IR significantly affects skeletal muscle, adipocytes, and liver tissue because of their high metabolic requirement (35, 36).

#### 1.5.4 Shared risk factors for T1DM and T2DM

**Figure 6:**
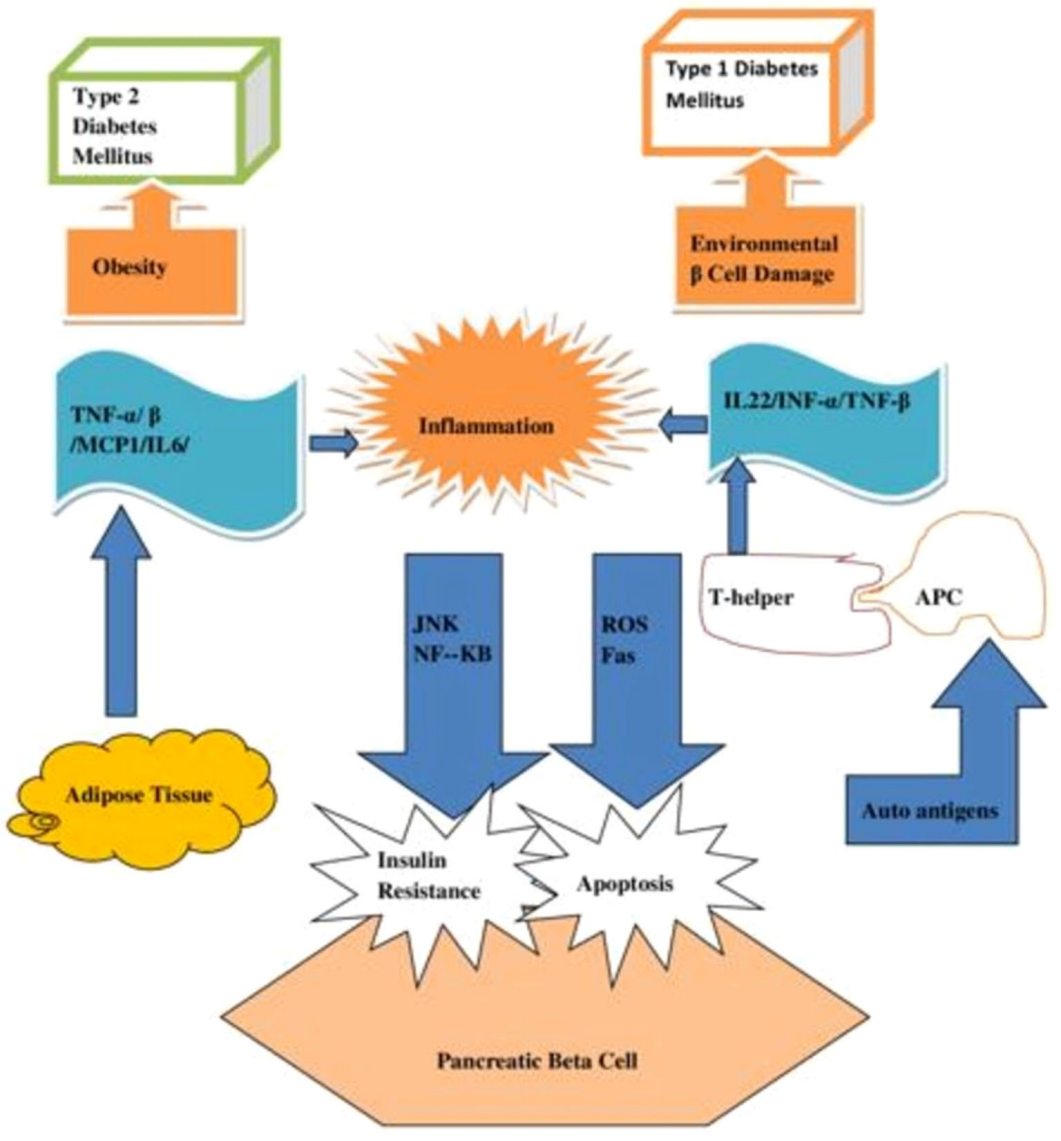
A diagram showing the common risk factor of inflammation for diabetes mellitus of both Types 1 and Type 2.

Although the risk factors for T1DM and T2DM differ significantly. An overlapped risk factor contributes to their likelihood. **Figure 6** shows common risk factors for T1DM and T2DM. Inflammation is a risk factor that both T1DM and T2DM share because it kills β-cells. T1DM patients with damaged β-cells emit auto-antigens, to which the T-helper was exposed via antigen-presenting cells (APC). Active T-helper cells create cytokines that increase inflammation, which in turn causes ROS and Fas to be released, which cause β-cell death. Similarly, adipose tissues in T2DM generate cytokines that trigger JNK and NF-κB pathways, which in turn intensify inflammation and impact insulin signaling in β-cells. APCs that deliver antigens, TNF-α and β (tumor necrosis factor α and β), MCP-1 (monocyte chemo-attractant protein-1), IL-6 (interleukin-6), IL-1β (interleukin-1β), and plasminogen-activator inhibitor-1 (PAI-1) (37).

### 1.6 Current international insights on DM complications

Although there has been a reported global increase in the prevalence of the illness, there are fewer obvious international patterns in complications. Regrettably, information regarding the patterns of diabetes-related complications is scarce, encompassing only a few dozen primarily affluent nations. The evolving landscape for LMICs remains indeterminate (38). Over the previous twenty years, high-income countries (HICs) have seen a decline in the annual rates of T2DM-related vascular complications and mortality; nonetheless, certain underrepresented and indigenous racial/ethnic population subgroups continue to bear a disproportionately high burden of these problems. There was a significant knowledge gap that has to be filled regarding the neurological, hepatic, ophthalmic, or renal issues related to DM (39, 40). Lack of data availability, representativeness, accessibility, timeliness, usability, and/or competence to process and analyze clinical and administrative data is frequently the causes of this. For this reason, the majority of DM complications estimates in LMICs originate from surveys where results are either self-reported or modeled using transition probabilities (i.e., relative risks of complications) derived from data from HICs (39).

### 1.7 Diagnosis of DM

Generally, according to the American Diabetes Association (ADA) Diagnostic Guidelines (**Table 1**), there are four common tests utilized for diagnosis of DM, namely: 1) Random (casual) plasma glucose tests; 2) Fasting plasma glucose tests; 3) Oral glucose tolerance tests (OGTT) and 4) glycated hemoglobin (HbA1C) test (6, 41).

**Table 1:** The ADA diagnostic guidelines: adapted from (6, 41).

Stages	Latent	Impaired Glucose Tolerance(IGT)	Diabetes
Criteria for Diagnosis	If two or more autoantibodies are present and the glucose level is normal	100–125 mg/dl for fasting blood glucose; 140–199 mg/dl for 2 hours during the OGTT; or 5.7–6.4% for HbA1C. 1)	Blood glucose measured during fasting: ≥126 mg/dl or 2 h blood glucose measured during OGTT: ≥200 mg/dl or random plasma glucose measured with symptoms of polyuria and weight loss: ≥200 mg/dl or HbA1C ≥6.5%.

IGT, Impaired Glucose Tolerance; OGTT, Oral glucose tolerance tests; HbA1C Glycated Hemoglobin.

## 2 Methodology: literature searching strategy

The search of works of literature was done utilizing search engines such as PubMed, Web of Science, Scopus, Science Direct, Wiley Online Library, Google Scholar, and Research get to compile the findings of lifestyle modification and new and innovative medications used globally to manage diabetes mellitus. We have conducted an extensive search using keywords/phrases such as “diabetes definition,” “pathophysiology of diabetes mellitus,” “signs and symptoms of diabetes,” “diagnosis of diabetes,” “major risk factors for diabetes” “nonpharmacologic management of diabetes,” “current therapeutic agent for diabetes,” “novel therapeutic agent,” and “targets for diabetes management” to find relevant papers addressing our goal. In addition, a thorough investigation is done on literature that is written in English and is sound scientifically.

## 3 Pharmacological treatment and management of DM

Treatment and management of DM is a universal health problem and successful treatment is yet to be discovered. It affects the human body at multiple organ levels thus making it difficult to follow a particular line of the treatment protocol. Consequently, it requires a multimodal approach including non-pharmacological and pharmacological management. Multifactorial treatment of DM can able to minimize both macro and microvascular complications (42).

### 3.1 Lifestyle modification for the management of both T1DM and T2DM

Food intake and exercise are the two main determinants of energy balance and are the cornerstones of DM management. Blood sugar levels must be regularly checked in people with T1DM. There are several ways to lower your risk of health issues, including creating a healthy food plan, getting regular exercise, and collaborating with the diabetes team to modify insulin therapy. Clinical investigations indicate that lifestyle modifications can lessen the risk of developing T2DM by delaying or preventing its onset (43, 44).

The risk can be lowered by about 58% in just three years. Individuals with IGT, impaired fasting glucose (IFG), or an HbA1C level of 5.76-6.4% are strongly encouraged to obtain dietary and activity advice, according to the ADA. On the other hand, individuals who have already received a diabetes diagnosis should adhere to the dietary suggestions made by a qualified dietician (45). A certified dietician should provide nutrition counseling to those with diabetes. When combined with other elements of diabetes management, nutrition therapy can lower HbA1C by 1-2% and enhance clinical and metabolic results. For diabetic individuals who are also overweight (obese), a treatment objective should be reducing calorie consumption to reach and maintain a healthier body weight. The distribution of macronutrients is variable within advised ranges and will rely on the preferences and goals of each individual’s therapy. There is a clinically significant improvement in glycemic control in individuals with T1DM and T2DM when low-glycemic-index carbohydrates are substituted for high-glycemic-index carbohydrates in mixed meals (46).

Aiming for moderate weight loss (≈7percentage of body weight) can help with blood glucose control, blood pressure and cholesterol management, and diabetes prevention and treatment. By controlling total calories and free carbohydrates in a well-balanced diet, weight loss is possible. Nonetheless, diabetic individuals who strictly follow a low-carb diet should be aware of potential adverse effects such as headaches, constipation, and hypoglycemia. To enhance glycemic control, other research has recommended consuming whole grains and complex dietary fiber. Research indicates that exercise, with or without a notable reduction in body weight, can enhance glycemic management (a reduction of 0.66% in the HbA1C level) and enhance patients’ overall quality of life (45, 47, 48).

Adults ≥18 years of age should, to reap the greatest benefits, participate in moderate-intense activity for at least 150 min a week (such as walking at a 15–20 min mile pace) or 75 min a week of energetic physical activity (such as running, aerobics) spread over at least three days per week with no more than 2 days without exercise. For individuals who are at least 18 years old,1 h of physical activity each day is sufficient (49, 50).

Additional lifestyle modifications that should be taken into account in the treatment plan for patients with diabetes include reducing sodium intake and moderate alcohol consumption (≤1 drink for women, ≤2 drinks for men), particularly in patients with comorbid conditions like hypertension, habitual tobacco use, and immunization deficiency (pneumococcal, hepatitis B, influenza, diphtheria, pertussis, tetanus, and tetanus). DM patients during should not consume alcohol while they are under treatment as it might cause potentially fatal hypoglycemia and coma, especially when fasting. Additionally, to effectively counteract the negative consequences of diabetes, patient education, counseling, and psychosocial support are crucial (50).

### 3.2 Current pharmacologic management of DM

Improving glucose control and lowering long-term consequences in T2DM are linked to early pharmacologic therapy beginning. Currently, it is treated using the following drug classes: insulin; biguanides; sulfonylureas; meglitinide derivatives; α-glucosidase inhibitors; thiazolidinediones; glucagon-like eptide–1-agonists; glucose-dependent insulinotropic polypeptide agonists; dipeptidyl peptidase iv inhibitors; selective sodium-glucose transporter–2 inhibitors are being employed as treatment regimens to manage DM across the globe. Each of these is covered in the manuscript’s next section (51, 52).

#### 3.2.1 Insulin therapy for the management of T1DM and T2DM

For all T1DM patients, insulin is the main course of treatment. When first diagnosed, patients with T1DM usually need to start with several daily injections. Typically, one or more daily separate injections of intermediate- or long-acting insulin are administered in addition to 0 to 15 minutes of fast-acting insulin or rapid-acting insulin analog. One can use two or three premade insulin shots every day. The target HbA1c should be < 7.5% (< 58 mmol/mol) for all children having T1DM, including preschool-age children (53).

It is recommended to start insulin therapy in T2DM patients in the following situations: if they have an acute illness or surgery; are pregnant; have glucose toxicity; in case of severe kidney or liver failure are not able to reach their goals with oral antidiabetic drugs, or require flexible therapy. When HbA1c is = 7.5% (=58 mmol/mol), insulin is considered as a monotherapy or in combination with oral agents to help T2DM patients reach their glycemic goals. When HbA1c is =10% (= 86 mmol/mol), insulin is needed for treatment, if diet, exercise, and other antihyperglycemic agents have been properly implemented to their fullest potential (53, 54).

#### 3.2.2 Biguanides for the management of T2DM

The first-line treatment for T2DM blood sugar reduction has been metformin, a biguanide medication. The FDA has authorized this medication. This medication improves glycemic management by altering the liver’s sensitivity to insulin. Nevertheless, there is little information available regarding the adverse effects of metformin, mostly from case reports. it’s worth noting that metformin may harm a patient’s ability to get a good night’s sleep by causing atypical nightmares and, in rare instances, lactic acidosis (55).

#### 3.2.3 Sulfonylureas for the management of T2DM

People with T2DM who are not extremely obese are frequently treated with second-line medications known as sulfonylureas. T2DM has been treated with sulfonylureas since the advent of tolbutamide in the 1950s. They are divided into two groups: first-generation (acetohexamide, tolbutamide, chlorpropamide, and tolazamide) and second-generation (glibenclamide, ligmepiride, gliclazide, glipizide, and gliquidone agents). The fundamental distinction between both generations is that the agents in the second generation are substantially more powerful than those in the first. Sulfonylureas are secretagogues of insulin that enhance the quantity of insulin produced by pancreatic β-cells, therefore reducing plasma glucose levels (56–58).

Their mechanism action is by directly obstructing ATP-sensitive K+ channels on islet cells, which increases the generation of insulin. They depend on the presence of a sufficient number of cells with a sufficient functional reserve, but they remain effective until they accomplish their intended goals whether taken alone or in conjunction with other anti-hyperglycemic drugs. Sulfonylureas’ primary acute side effect, is a higher incidence of hypoglycemia, particularly in elderly patients with impaired renal function, hepatic dysfunction, poor oral intake, calorie restriction, alcohol abuse, and other disorders (56, 59).

#### 3.2.4 Meglitinide derivatives for the management of T2DM

People with T2DM can better regulate their blood sugar levels by combining a healthy diet and exercise regimen with the insulin secretagogues repaglinide and nateglinide, generally known as “glinides.” Meglitinide derivatives, as a monotherapy or in conjunction with metformin, can help adults with T2DM improve their glycemic control in addition to nutrition and exercise (60, 61). The regulation of insulin synthesis by pancreatic β-cells involves cell membrane potential, which is determined by the inverse relationship between extracellular glucose levels and potassium channels’ activity that are sensitive to adenosine triphosphate. Glucose transporters 2 transfer (GLUT_2_) extracellular glucose into the cell. The cell uses and stores adenosine triphosphate (ATP) as energy after breaking down glucose as it enters the body. They increase the release of insulin by inhibiting ATP-sensitive potassium channels, which depolarize β-cells, and opening calcium channels, which let calcium in. Production of insulin is stimulated by elevated calcium in the cells levels (61, 62).

#### 3.2.5 α-glucosidase inhibitors (AGIs) for the management of T2DM

Among the oral AGIs used to treat diabetes are voglibose, miglitol, and acarbose. Inhibitors of α-glucosidase stop the small intestine from absorbing carbohydrates. They obstruct the enzymes that change complicated, non-absorbable carbohydrates into simple, absorbable ones through competitive inhibition. These comprise the following enzymes: isomaltase, maltase, sucrase, and glucoamylase. They delay the absorption of carbohydrates, which lessens an increase in blood sugar levels after meals by about 3 mmol/l (reduced postprandial glucose). The drug in this class that is most frequently used and researched is acarbose. α-amylase, sucrase, maltase, and dextranase are all inhibited by acarbose, however, it is more effective against glucoamylase. On the other hand, it does not affect the lactase β-glucosidase. These drugs are eliminated by feces, have a low absorption rate from the stomach, and have restricted bioavailability. Conversely, miglitol goes entirely through the kidneys and bypasses the stomach. While miglitol and voglibose do not undergo intestinal metabolism, acarbose does. For people who have a low glucose tolerance, in particular, they are therefore advantageous (63, 64). A doctor may recommend AGIs to a patient diagnosed with T2DM if they observe that their blood sugar tends to increase after meals. Moreover, the doctor might recommend adding an AGIs to their diabetic regimen if the patient is on medication for excessive blood sugar (65).

#### 3.2.6 Thiazolidinediones (TZDs) for the management of T2DM

TZDs (troglitazone, pioglitazone and rosiglitazone) are also known as glitazones work by sensitizing insulin to T2DM. Since their introduction in the late 1990s, TZDs have been utilized extensively because of their therapeutic benefits in treating insulin resistance and maintaining glycemic control. Troglitazone is the first TZDs drug that is approved by the FDA. However, it was taken off the market after 3 years as some patients’ experienced serious liver toxicity. Right now, pioglitazone and rosiglitazone are the only TZDs medications available in the market for clinical use. TZDs are also recognized to possess anti-inflammatory and anti-cancer characteristics (66).

There are no pharmacologic treatments that particularly treat insulin resistance other than TZDs. They are widely established that TZDs reduce the cardiovascular risk factors linked to insulin resistance. However, TZDs uses have been restricted because of worries about potential side effects and safety concerns. For instance, pioglitazone lowers myocardial infarctions (MI) and ischemic strokes, according to recent findings. The capacity of clinicians to choose patients who would experience few to no major side effects is enhanced by new information regarding TZDs-mediated congestive heart failure, bone fractures, and edema (67).

Patients with T2DM benefit from TZDs because they lower glycemia, dyslipidemia, and insulinemia. By initiating the nuclear receptor peroxisome proliferator-activated receptor-γ (PPAR-γ), they modify the expression of genes related to the homeostasis of glucose and lipids. Insulin sensitivity is increased when PPAR-γ is stimulated through many pathways. It does this through three different means: 1) it increases the expression of GLUT_4_; 2) it controls the release of signaling molecules obtained from adipocytes that influence muscle’s sensitivity to insulin; and 3) it induces apoptosis in adipose tissue, it causes the formation of adipocytes that are smaller and more flexible. Pancreatic β-cell activity is enhanced by TZDs by influencing the lipotoxicity of free-fatty- acids on islet cells of the pancreas. Recent approval for European commercial use has been granted to two TZDs: pioglitazone and rosiglitazone (68).

#### 3.2.7 Peptidyl peptidase-4 inhibitor (DPP-4 inhibitors) for the management of T2DM

DPP-4 Inhibitors, also known as “gliptins” like sitaglibtin, saxagliptin, linagliptin, and alogliptin, progressively replace sulfonylureas to manage T2DM in numerous countries. The three main advantages of these drugs are: 1) not associated with hypoglycemia or weight gain; 2) have a good safety profile; and; 3) used as an alternative to drugs such as metformin and sulfonylureas when fail. Their mechanism of action includes: increasing the mass and function of pancreatic β-cells; increasing insulin sensitivity in liver, muscle, and adipose tissue; decreasing dyslipidaemias; increasing fat oxidation and cholesterol efflux; lowering hepatic triglyceride synthase, decline *de novo* lipogenesis; postponing the time for stomach emptying and promoting satiety, have anti-inflammatory and antiatherogenic impacts, and improves endothelial function and reduces vascular stiffness (69). DPP-4 inhibitors are employed either as an add-on drug therapy when metformin (a biguanide), or sulfonylurea is inadequate or as monotherapy in individuals who should not be taking those medications or who are intolerant to them (70).

#### 3.2.8 Glucagon-like peptide receptor agonist for the management of T2DM

Glucagon-like peptide-1 (GLP-1) receptor agonists also referred as incretin mimetics, or GLP-1 analogs like lixisenatide, liraglutide, albiglutide, exenatide, dulaglutide, and semaglutide are alarmingly used in combination with basal insulin to optimize glycemia, reduce weight, and optimize insulin dose requirements. While exenatide is the first incretin mimetic licensed to be used in patients with T2DM, liraglutide is the preferred GLP-1 receptor agonist that can be used among those who have impaired renal function. Furthermore, high-dose liraglutide is FDA-approved as a pharmacologic treatment for obesity or can be prescribed to overweight patients with comorbidities. The benefits of this form of therapy to treat T2DM include 1) delayed gastric emptying, and 2) inhibiting the production of glucagon from pancreatic α- cells (71–73).

#### 3.2.9 Sodium glucosinolate co-transporter 2 inhibitor for the management of T2DM

Sodium glucosinolate co-transporter 2 (SGLT-2) inhibitors (empagliflozin and dapagliflozin) are the most beneficial as an adjunct medication in addition to metformin in patients with a history of cardiovascular or renal disease that needs further HbA1c reduction. Due to their ability to lower the renal glucose threshold and raise urine glucose excretion, these medicines lessen hyperglycemia. The subsequent lowering of glucotoxicity enhances the sensitivity of tissue insulin and pancreatic β-cells to glucose. SGLT-2 inhibitors can also able to lower the body weight of individuals with obesity (74–76).

## 4 Novel and emerging therapeutic agents and/or targets for the management of both T1DM and T2DM

Despite the development of many well-known and recently created drugs, none of them are yet able to fully address the medical requirements of patients with diabetes. To control and cure diabetics, it is critical to investigate new, safe, and effective medications and molecular targets. This is because diabetes is becoming more common and has few therapeutic targets. Instead of reversing diabetes, the current treatment plans for DM just slow down the disease’s progression. If applied earlier on, their effectiveness could (in theory) be greatly increased. Therefore, the primary emphasis of scientists worldwide is finding new therapeutic agents for diabetes, either by improving the effectiveness and tolerability of currently available medications or by developing novel antidiabetic therapies soon. In the section that follows, we discussed a few targets and/or treatment alternatives that we thought showed promise in the following section, keeping in mind that there is little to no worldwide focus on the effective management of both T1DM and T2DM.

### 4.1 Vitamin C and vitamin E for the management of T2DM

Supplementing with vitamin C (ascorbic acid) may be a useful adjunct therapy for managing diabetes in individuals, according to emerging data because of its free radical scavenging, antioxidant and anti-inflammatory properties. Vitamin C supplements can lower some indicators of OS in T2DM, which is consistent with its biological effects of redox modulation. Vitamin C supplementation dramatically lowers plasma malondialdehyde (toxic and biological marker for OS) levels in individuals with T2DM (77, 78). Similarly, Vitamin E consumption is effective in lowering insulin resistance and HbA1c in DM Patients. Furthermore, patients have experienced a decrease in their fasting blood glucose levels after receiving brief vitamin E therapies (79).

### 4.2 Mineralocorticoid receptor antagonists (MRA) for the management of T2DM

MRA are a novel class of drugs that were developed in response to the medical community’s need for a safer, more efficient way to treat patients with diabetic kidney disease (DKD) while simultaneously safeguarding their hearts and kidneys. The kidney and heart responded favorably to the nonsteroidal MRA finerenone in a variety of individuals with CKD and T2DM. Finerenone’s long-term effects on renal and cardiac morbidity and mortality endpoints, along with the anti-hypertensive efficacy of esaxerenone, broaden the range of treatments available to patients with DKD (80).

### 4.3 Proprotein convertase subtilisin/kexin-9 inhibitors (PCSK9 inhibitors) for the management of T2DM

A hepatic enzyme proprotein convertase subtilisin/kexin-9 regulates circulating low-density lipoprotein cholesterol levels by binding to low-density lipoprotein receptors, thereby promoting their degradation. PCSK9 inhibitors like evolocumab and inclisiran have been shown to lower low-density lipoprotein cholesterol levels and to reduce the rate of cardiovascular events among patients with established cardiovascular disease accompanied with T2DM (81, 82).

### 4.4 Protein tyrosine phosphatase-1B inhibitors (PTPase-1B) for the management of T2DM

Insulin initiates its stimulatory action on glucose metabolism by causing the phosphorylation of three tyrosine residues on the insulin receptor. Because insulin receptor tyrosine phosphorylation is reduced in people with T2DM, it makes sense to increase tyrosine phosphorylation with PTPase-1B, a target in the signaling pathway of insulin. Asperentin B is a novel inhibitor of PTPase-1B and a vital alternative in T2DM therapy (83).

### 4.5 Dual glucagon-like peptide-1 (GLP-1) receptor agonist and glucose-dependent insulinotropic polypeptide (GIP) receptor for the management of T2DM

The intestinal hormone GLP-1 plays a wide range of physiological activities during the postprandial state. It stimulates insulin secretion during euglycemia and hypoglycemia, and it stimulates glucagon secretion besides, glucose-dependent insulinotropic polypeptide increases tri-acyl glycerol (uptake in adipose tissue and decreases bone resorption (84). Postprandial insulin secretion is stimulated by the combined action of the gut-derived incretin hormones GIP and GLP-1. These hormones are released following meal consumption. Moreover, at low glucose levels, GIP works glucagonotropically, but GLP-1 suppresses glucagon production when plasma glucose concentrations are higher than typical fasting levels. A dual incretin receptor agonist that co-activates GLP-1 and GIP receptors has been shown in a clinical trial involving overweight/obese patients with T2DM to produce significant improvements in glycemic control (mean HBA1c reduction of 1.94%) and massive weight loss (mean weight loss of 11.3 kg) after 26 weeks of treatment with the highest dose (15 mg/weekly) (85). Tirzepatide is a novel pharmacological class that acts as GLP-1/GIP receptor agonist. As the first and only FDA-approved dual GLP-1/GIP medication, tirzepatide helps patients lose weight by promoting hormones that make them feel fuller for longer. In addition, tirzepatide lowers systolic blood pressure in adults who are obese, which can reduce the risk of hypertension and cardiovascular issues (86–88). Tirzepatide operates similarly to GLP-1 receptor agonists but boosts the incretin impact synergistically to help manage blood glucose levels and promote weight reduction. It mimics the GLP-1 and GIP hormones that the intestine naturally secretes after a meal, encouraging insulin release. It also lowers appetite by shortening the time it takes for the stomach to empty and signaling satiety by interacting with GLP-1 receptor-containing areas of the brain (89).

### 4.6 Dopamine 2 receptor agonist (D2 receptor agonist) for the management of T2DM

The D2 receptor agonist (bromocriptine and cabergoline), were originally intended to treat Parkinson’s disease, pituitary tumors, and prolactinomas, however, they also have effects on glucose metabolism. D2 receptor agonist significantly lowered fast blood sugar and HbA1c levels without causing severe negative effects (90). Bromocriptine is a quick-release sympatholytic dopamine D2 receptor agonist, which is useful for T2DM therapy. It is a postprandial insulin sensitizer, hence, improves glycaemic control in T2DM when glycemia is poorly controlled by metformin plus basal-bolus insulin, or individuals on high-dose basal-bolus insulin (91).

### 4.7 Bile acid sequestrant and statin for the management of T2DM

Bile acids are nutrient sensors and metabolic integrators that regulate lipid, glucose, and energy homeostasis (92). Bile acid exposure in both the small and large intestines induces GIP polypeptide-1 secretion, modulates the composition of gut microbiota, and reduces postprandial blood glucose excursions in humans with and without T2DM. Colesevelam is a typical bile acid sequestrant that is approved to treat T2DM with hyperlipidemia. It improves glycaemic control through hepatic microRNAs (miRNAs) 182–5p. Moreover, it increases the level of incretins and glucose-dependent insulin secretion (93, 94). T2DM is a significant risk factor for cardiovascular illnesses, thus there is a close relationship between the two conditions. Furthermore, T2DM frequently occurs before cardiovascular illnesses do.

The incidence of cardiovascular disease can be effectively decreased by statin therapy (95). Patients with comorbidity of diabetes and cardiovascular risk should start statin therapy with target low-density lipoprotein cholesterol of less than 100 mg/dl, according to the ADA’s standards. Patients with diabetes and cardiovascular disease may also want to aim for a target low-density lipoprotein of less than 70 mg/dl. Statins appear to minimize major cardiovascular events in both primary prevention and very-high-risk individuals, regardless of the obtained baseline and post-therapy low-density lipoprotein levels, at least partially. If a diabetic patient’s low-density lipoprotein cholesterol appears to be within normal ranges, statins should often be prescribed in a fixed-dose manner (96, 97).

### 4.8 Amylin analogues for the management of T2DM

Amylin (islet amyloid polypeptide), commonly known as a diabetes-associated peptide, is secreted in DM patients’ islets of Langerhans in a 1:100 ratio with insulin. Compared to insulin monotherapy, patients with T1DM who received amylin analog in addition to insulin experienced a greater reduction in pre-prandial hyperglycemia and a corresponding decrease in glucagon levels. After a meal, insulin-producing β-cells co-release amylin analogs, which seem to work in concert with insulin. It has been demonstrated that amylin analogs, such as pramlintide, dramatically lower body weight, HbA1c levels, and even insulin dosage (98, 99).

### 4.9 Inorganic nitrate/nitrite for the management of T2DM

Research from both *in vitro* and/or *In vivo* models suggests that inorganic nitrate/nitrite may serve as a substitute for endogenous nitric oxide (NO), improving glucose absorption and the signaling route for insulin while reducing insulin resistance and the problems associated with diabetes. In diabetes, dietary nitrate/nitrite may also mitigate other impaired NO-dependent processes, such as blood pressure regulation, vascular function, mitochondrial biogenesis, energy balance, adipose tissue metabolism, and lipid and lipoprotein metabolism (100).

### 4.10 Gamma amino butyric acid (GABAergic systems) as a novel target for management of T1DM

**Figure 7:**
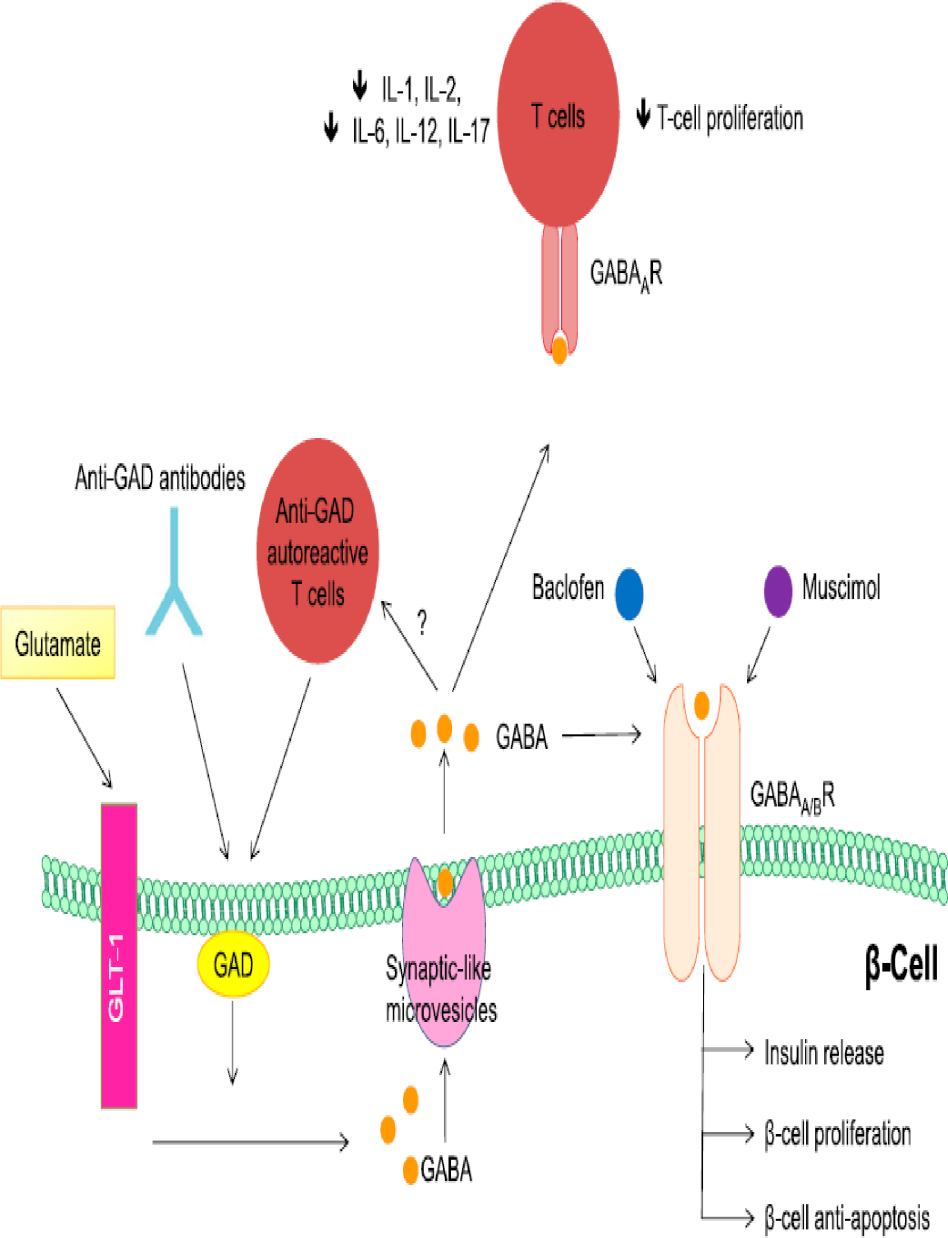
Regenerative and role GABA in pancreas beta cell: Adapted From (101).

Pancreas β-cells express gamma amino butyric acid receptor A/B (GABA_A_/_B_Rs) and activation of GABA receptors leads to Ca^2+^-influx stimulating or activation of PI3K/Akt/PKB pathway resulting in increased insulin release promotes pancreatic β-cells proliferation and inhibits apoptosis. Thus, GABA regulates both the survival and regeneration of human pancreatic β-cells. Baclofen and muscimol, both activate GABA_B_R on pancreas β-cell. Activation of the GABA_A_ receptor in diabetogenic immune cells (CD4, CD8, B-cells, auto-reactive T-cell, monocytes) leads to the protection of β-cells by decreasing the production of inflammatory cytokines and inhibiting or reducing T-cell proliferation (**Figure 7**) and inhibits anti-GAD auto-reactive T-cell (101–104).

### 4.11 Probiotic: an emerging approach for prevention and management of both T1DM and T2DM

Probiotics have become popular as dietary supplements among the public and medical community due to their potential importance in promoting health, notably the treatment of both types of diabetes. Probiotics from *Bifidobacteria* (including *Bifidobacterium infantis, Bifidobacterium longum*, and *Bifidobacterium breve*) and *Lactobacilli* (including *Lactobacillus acidophilus, Lactiplantibacillus plantarum, Lactobacillus bulgaricus, and Lactobacillus delbrueckii subsp*. that were taken orally showed encouraging antidiabetic effects. Furthermore, they have demonstrated that exposure to *Lactiplantibacillus plantarum* and *Lactobacillus genus* can delay or prevent autoimmune DM in healthy people.

Probiotics may boost incretins, decrease endoplasmic reticulum stress, decrease systemic lipopolysaccharide levels, improve peripheral insulin sensitivity, and preserve the integrity of the gut. Probiotics may also improve glucose tolerance, control lipid metabolism, boost antioxidant levels, alter the composition of long-chain fatty acids in the gut microbiota, and increase antioxidant status. Furthermore, probiotics diminish the inflammatory, autoimmune, and OS responses.

*Bifidobacterium lactis* and *L. plantarum* are probiotic bacteria that can help reduce the detrimental effects of high-fat meals and even modulate immune responses brought on by inflammatory illnesses*. Lacticaseibacillus rhamnosus*, when mixed with *B*. *lactis* and *Lactobacillus gasseri*, has been demonstrated to inhibit weight gain in humans, specifically fat tissue mass adiposity, which strengthens the efficacy of probiotics in diabetes (105–107). Because they can alter gut flora and help prevent childhood obesity, diabetes, and autoimmunity, probiotics are becoming a well-liked therapy alternative for both T1DM and T2DM (108, 109). The gut flora mediates three pathways connected to the pathogenesis of obesity and diabetes: low-grade inflammation, raised blood levels of lipopolysaccharides (endotoxemia), and increased energy harvest. One possible target for the fight against obesity and diabetes has been suggested: modifications to the gut flora. Probiotic-supplemented fermented milk products (yogurt, enhanced with *Lactobacillus* and *Bifidobacteria*) prevented streptozotocin-induced diabetes in animal models and greatly reduced diet-induced IR. Probiotics are a novel class of gut flora modulators that may be helpful in the management and prevention of diabetes and obesity. By enhancing the antioxidant system, probiotic has also been found to prevent the onset of diabetes and its aftereffects. Understanding the mechanisms underlying the therapeutic advantages of probiotics is important, but so is figuring out which strains, dosages, and treatment durations work best. Additional study is necessary to answer these questions (110–113).

### 4.12 Fructose-1, 6-bisphosphatase (FBPase) inhibitors for management of T2DM

FBPase, a rate-regulating gluconeogenesis enzyme, has emerged as a key target in T2DM therapy due to the recognized importance of excessive endogenous glucose synthesis in the hyperglycemia that defines the condition. Since gluconeogenesis is known to regulate glucose overproduction, FBPase has emerged as a viable molecular target. However, none of the drugs on the market today even directly lowers gluconeogenesis. Since most of the existing antidiabetic drugs primarily target insulin resistance or insufficiency rather than directly or efficaciously reducing gluconeogenesis, inhibitors of FBPase have been expected to address an unmet medical need (114).

Strong and specific FBPase inhibitors that target the enzyme’s allosteric region were found despite several obstacles by employing a structure-guided design approach that began with the natural inhibitor adenosine monophosphate. In diabetic rodents, the prodrug CS-917 produced a promisingly lower rate of gluconeogenesis and endogenous glucose production. Clinical tests of CS-917 conducted later on in individuals with T2DM showed a good safety profile and clinically significant decreases in fasting glucose levels. The potential for this unique class of antidiabetic medicines to provide long-term, safe glycemic control will be investigated in future studies of MB07803, a second-generation FBPase inhibitor with better pharmacokinetics (114, 115).

### 4.13 Gene therapy for management of T1DM

Gene therapy is a therapeutic approach in which a person’s DNA is modified in various ways to treat, and in rare cases, even cure a particular condition. A gene that causes the disease can be silenced if it’s not functioning properly, replaced with a healthy copy, or added to the body to assist treat a condition. Research on gene therapy is now and in the future focused on common conditions such as cancer, infectious diseases, and hereditary/autoimmune illnesses including T1DM. Gene therapy is in its infancy and is only now available in clinical trials, but the benefits of this treatment are starting to become more apparent based on the existing data. The many forms of gene therapy include plasmid DNA, bacterial, viral, and human gene-editing technologies, as well as patient-derived cellular gene therapy products (116).

Research conducted on animals (preclinical) shows that gene therapy can be applied in various ways and preserves pancreatic β-cells, which optimizes insulin secretion levels. To help regenerate β-cells, targeted viral vector transduction or expression of genes, such as lentivirus or adenovirus, and mRNA-transfected T-cells that target insulin-reactive CD8 T cells, can help prevent T1DM. Another method is to create insulin-producing cells in the liver by gene transfer using anti-TCRβ mAb and Ngn3-Btc (116).

### 4.14 Stem cell immunotherapy for management of T1DM

The unique properties of pluripotent cells are being used by the quickly expanding field of stem cell immunotherapy to revolutionize healthcare. From autoimmune diseases to cancer, stem cell immunotherapy has vast and transformative promise. This article examines the principles of stem cell immunotherapy and some of its potential applications in, a promising new treatment avenue. The four categories of contemporary T1DM immunotherapies are; 1) stem cell therapies; 2) β- cell therapies; 3) antigen-independent techniques; and 4) antigen-dependent strategies. Since mesenchymal stem cells (MSCs) play a key role in tissue repair and regeneration, numerous trials are currently trying to test the use of MSCs from various sources for the treatment of T1DM (117, 118). MSC is a population of stem cells that exhibits progenitor cell characteristics of self-renewal and differentiation (119). More accurate and targeted treatment approaches based on MSC have increased the possibility of curing type T1DM. To cure diabetes for good, this cell-based therapy aims to produce functional β-cells that secrete insulin. Stem cell-based therapies have a great deal of therapeutic potential because of their innate ability to control immunity and regenerate. In this post, we have covered in detail the role that MSCs play in treating diabetes (120).

There are established protocols for differentiating pluripotent stem cells into either fully differentiated β-cells or pancreatic progenitors. MSCs are widely accessible and come from several tissues. Through controlled differentiation, they can develop into cells that produce insulin. Empirical data has demonstrated that the transplantation of allogenic MSC-derived insulin-producing cells results in a mild allogeneic response that does not affect the cells’ ability to function. The immune-modulatory properties of the MSC subset that did not develop into cells that produce insulin explain this (121). Exosomes produced from naive MSCs have been applied in the experimental setting to cure diabetes in mice, with differing degrees of success. Several mechanisms, including the reduction of insulin resistance, the promotion of autophagy, and the increase in the T-regulatory population, have been proposed to account for their beneficial benefits (122).

Additionally, several *in vitro* and *in vivo* investigations have demonstrated that the differentiation of embryonic stem cells into cells resembling insulin secretagogue activity leads to an enhancement of glucose absorption and metabolism following ESC transformation. Likewise, the possibility of repairing the injured pancreas in diabetic patients was demonstrated by intravenous injection of embryonic-like stem cells in mice with pancreatic necrosis (116, 123, 124).

### 4.15 Unfolding monoclonal antibodies for management of autoantibodies in T1DM

It is commonly known that autoantibodies have the capacity to be extremely dangerous antibodies that attack the host by attaching to self-antigens, leading to serious autoimmune diseases. However, the identification of autoantibodies against a variety of disease-associated antigens has made it possible for them to be effectively used as crucial instruments in the diagnosis, prognosis, and therapy of disease (125). For the purpose of preventing and treating T1D in clinical settings, immunotherapies that specifically restore persistent β-cell-specific self-tolerance are still needed. One tactic to target immune cell populations that cause autoimmune-driven pathology is the use of monoclonal antibodies, which have been shown to be clinically safe and to have varying degrees of efficacy in modulating autoimmunity, including T1D (126). Monoclonal antibodies have been shown to have two primary targets that are effective in both animal models and human research. These targets are as follows: B-cell-tracking agents: Anti-CD20 monoclonal antibody rituximab is known to deplete B-cells; T-cell targeting agents: The ϵ chain of the CD3 receptor on the surface of T cells, formerly referred to as muromonab-CD3, is now the most promising therapeutic target in changing the course of T1DM. Anti-CD3 medication can cause diabetic remission in T1DM models, according to research on animals. The primary impact is linked to the activation of regulatory T lymphocytes and immune-suppressive cytokines such transforming growth factor-β. Research indicates that the most effective treatment for T1DM progression is telplizumab, a humanized anti-CD3 monoclonal antibody. According to a number of human clinical trials, teplizumab therapy is a powerful means of delaying the decrease in C-peptide production and can support the maintenance of β-cell function (126, 127).

### 4.16 Phytomedicine: an indispensable approach in complementary and alternative medicine for DM

The WHO as being used for medical purposes across the globe lists approximately 21,000 botanicals, or plants and herbs. Phytomedicine is a cost-effective alternative to synthetic medications for treating DM, offering fewer side effects, longer-lasting benefits, and improved patient compliance. As a result, medicinal plants have been a valuable source of therapeutic agents, and many efforts have been made over the years to use these remedies to effectively manage DM. Despite the fact that over 400 plant species have been found to have hypoglycemic action in the literature, the search for new, natural anti-diabetic drugs is still interesting (128–130).

Plant secondary metabolites are abundant in bioactive chemicals that have numerous positive health impacts on both humans and animals. Numerous types of phytochemicals can be found in plant-based diets, such as vegetables, seeds, fruits, grains, legumes (131). Since ancient times, medicinal plants, which are abundant in terpenoids, steroids, polyphenols, tannins, alkaloids, carbohydrates, and flavonoids, have offered substantial health advantages. Many ailments and disorders can be treated with these phytochemicals, frequently with less adverse effects than traditional Western therapy. There are about 4,000 distinct kinds of flavonoids, a common class of phytochemicals found in nearly all plants. Flavonoids are part of the class of nutraceuticals that have been demonstrated to improve insulin sensitivity, manage hyperglycemia in diabetics, and govern metabolism. Strong antioxidant, antiviral, and anti-inflammatory qualities are exhibited by flavonoids and carotenoids, which comprise 60% of all polyphenols and are present in fruits and vegetables in high concentration. These substances’ antioxidant properties and bioactive ingredients have been clinically demonstrated to offer protection against a number of ailments, such as diabetes, cancer, heart disease, and kidney problems (132–134).

Phytomedicine plants are becoming useful in complementary and alternative medicines for treating DM and its complications because of the phytochemicals’ synergistic effects. It has been shown that a number of medicinal herbs and their preparations function at important glucidic metabolic sites (135–137). In the subsequent section, we have focused to point out about those medicinal plants that have great scientific anti-diabetic properties in *in-vitro* and *iv-vivo* models.

Although several phytomedicines are employed for DM management world-wide, the most recognized and scientifically validated includes*; Phyllanthus amarus* Schumach. & Thonn., *Momordica charantia* L, *Withania somnifera* L, *Morus alba* L., *Allium sativum* L., *Moringa oleifera* Lam., *Ocimum sanctum* L, *Eugenia jambolana* Lam., *Aloe vera* L, *Pterocarpus marsupium* Roxib., *Tinospora cordifolia* (Thynb.) Miers, *Phaseolus vulgaris* L*., Cinnamomum zeylanicum* J.Presl*, Trigonella foenum-graecum* L*., Zingiber officinale* Rosc*., Gymnema sylvestre* R.Br., *Trigonella foenum-graecum* L., *Panax ginseng* C.A.Meyer, *Citrullus colocynthis* (L.) Schrad., *Opuntia streptacantha* Lam., *Ficus bengalensis* L., *Salacia oblonga* Wall., *Salacia Reticulate* Wight, *Silybum marianum* (L.) Gaertn, *Panax quinquefolius* L, and *Panax ginseng* C. A. Mey. Herbals/botanicals have demonstrated potent anti-diabetic effects via a variety of mechanisms, such as enhancing insulin sensitivity, stimulating insulin secretion, blocking the absorption of carbohydrates, and reducing OS; the inhibition of α-glucosidase enzyme and the generation of advanced glycation products, the transcription of GLUT-4 and PPAR, and the presence of antioxidants. For instance, *M. charantia* stimulates insulin resistance and improves glucose absorption, whereas *G. sylvestre* secretes more insulin and helps to rebuild pancreatic β-cells. *W. somnifera* and *M. alba* are known to reduce OS and inflammation in addition to controlling glucose levels (128–130, 135–140).

Besides, botanical dietary supplements have been extensively researched for their potential to manage or prevent DM and its complications. The use of dietary supplements in people with diabetes continues to be a topic of debate, largely due to the scarcity of well-designed clinical trials. Many existing studies are inconclusive, often because of small sample sizes, differences in participant characteristics, variations in supplement formulations and dosages, and inconsistent research durations and outcomes. Consequently, there is insufficient evidence to definitively support the beneficial effects of most supplements on DM or its complications. *A. vera, M. charantia, C. cassia, T. foenum-graecum, G. sylvestre, P. ginseng, and P. quinquefolius* are a few of the plants that are often used with stronger evidence as dietary supplements for the management of DM in clinical research around the world. However, further research is urgently required to determine whether the other herbal and botanical remedies are also effective at treating DM-related symptoms, as well as which particular compounds have the greatest potential to be developed into safe and useful pharmaceuticals for clinical use. This research should utilize larger sample sizes, distinct participants, comparable supplement and dosage amounts, and a consistent length of study in large-scale clinical settings (140–142).

## 5 Conclusions and future prospects

DM is gaining a lot of attention these days due to its negative impacts on the global population. DM still has no known cure, despite being one of the earliest disorders in human history to be recognized. Due to the condition’s deadly effects on human life, a global search for a remedy for all age groups has been conducted. To experience and preserve a healthy and sound life, regular DM monitoring and management are required. Lifestyle modifications (healthy diet, regular exercise, aiming for moderate weight loss, consuming moderate amounts of sodium and alcohol, etc.) and the current anti-diabetic medications (insulin, biguanides, sulfonylureas, meglitinides derivatives, α-glucosidase inhibitors, thiazolidinediones, agonists of glucagon like peptide-1, glucose-dependent insulinotropic polypeptide agonists, inhibitors of dipeptidyl peptidase IV (DPP-4), sodium-glucose transporter–2 inhibitors are globally implemented to manage DM. However, these interventions have not satisfied patient medical needs due to their low efficacy (inability to cure), intolerability, potential for drug interactions, and a challenge for ongoing monitoring. Besides, the current treatments for both T1DM and T2DM focus on lowering blood glucose levels and easing the symptoms of any associated problems. For this reason, it is essential to enhance the profile of already available medications, look for and apply innovative approaches that require multimodal approaches for both T1DM and T2DM management and prevention. Vitamin C and Vitamin E, mineralocorticoid receptor antagonists, proprotein convertase subtilisin/kexin-9 inhibitors, protein tyrosine phosphatase-1B inhibitors, glucose-dependent insulinotropic polypeptide receptor and glucagon-like peptide-1 receptor agonist, amylin analogues, inorganic nitrate/nitrite, probiotics, gene therapy, stem cell immunotherapy, fructose-1, 6-bisphosphatase inhibitors, monoclonal antibodies, and targeting gamma amino butyric acid, phytomedicine, are novel and emerging strategies that are gaining attention for comprehensive and reliable management of both T1DM and T2DM. Nevertheless, more investigation is still necessary because none of them can completely reverse, stop, or even cure the long-term effects of diabetes mellitus. Additionally, new understandings of the mechanisms underlying these problems extend beyond a glucose-centric viewpoint to include problems with unseen organs. The development of more effective therapeutic approaches is therefore the main goal of DM management and control, while also taking into account the disorder’s potentially debilitating and financially detrimental repercussions.

## References

[1] Rodriguez-Saldana J. The diabetes textbook: Clinical principles, patient management and public health issues. London (global); Berlin (corporate); New York City (sales): Springer Nature (2023).

[2] Sidahmed S Geyer S Beller J. Socioeconomic inequalities in diabetes prevalence: the case of South Africa between 2003 and 2016. BMC Public Health. (2023) 23:324. doi: 10.1186/s12889-023-15186-w 36788553 PMC9926686

[3] Tattersall RB Matthews DR. The history of diabetes mellitus. Textbook Diabetes. (2024), 1–21. doi: 10.1002/9781444324808.ch1

[4] Punthakee Z Goldenberg R Katz P. Definition, classification and diagnosis of diabetes, prediabetes and metabolic syndrome. Can J Diabetes. (2018) 42:S10–S5. doi: 10.1016/j.jcjd.2017.10.003 29650080

[5] Bishu KG Jenkins C Yebyo HG Atsbha M Wubayehu T Gebregziabher M. Diabetes in Ethiopia: A systematic review of prevalence, risk factors, complications, and cost. Obes Med. (2019) 15:100132. doi: 10.1016/j.obmed.2019.100132

[6] American Diabetes Association. 2. Classification and diagnosis of diabetes: standards of medical care in diabetes—2020. Diabetes Care. (2020) 43:S14–31. doi: 10.2337/dc20-S002 31862745

[7] Leslie RD Palmer J Schloot NC Lernmark A. Diabetes at the crossroads: relevance of disease classification to pathophysiology and treatment. Diabetologia. (2016) 59:13–20. doi: 10.1007/s00125-015-3789-z 26498592

[8] American Diabetes Association. Standards of medical care in diabetes—2014. Diabetes Care. (2014) 37:S81–90. 2014; 37 (Suppl. 1): S14–S80 Diagnosis and Classification of Diabetes Mellitus. Diabetes Care. doi: 10.2337/dc14-S014 24357215

[9] Toren E Burnette KS Banerjee RR Hunter CS. Tse HM. Partners in crime: beta-cells and autoimmune responses complicit in type 1 diabetes pathogenesis. Front Immnol. (2021) 12. doi: 10.3389/fimmu.2021.756548 PMC8529969 34691077

[10] Roep BO Thomaidou S van Tienhoven R Zaldumbide A. Type 1 diabetes mellitus as a disease of the β-cell (do not blame the immune system)? Nat Rev Endocrinol. (2021) 17:150–61. doi: 10.1038/s41574-020-00443-4 PMC7722981 33293704

[11] Baynes H. Classification, pathophysiology, diagnosis and management of diabetes mellitus. J Diabetes Metab. (2015) 6:1–9. doi: 10.4172/2155-6156.1000541

[12] Frommer L Kahaly GJ. Type 1 diabetes and associated autoimmune diseases. World J Diabetes. (2020) 11:527–39. doi: 10.4239/wjd.v11.i11.527 PMC7672792 33269064

[13] Röder PV Wu B Liu Y Han W. Pancreatic regulation of glucose homeostasis. Exp Mol Med. (2016) 48:e219–e. doi: 10.1038/emm.2016.6 PMC4892884 26964835

[14] Tarabra E Pelengaris S Khan M. A simple matter of life and death-the trials of postnatal beta-cell mass regulation. Int J Endocrinol. (2012) 2012. doi: 10.1155/2012/516718 PMC3346985 22577380

[15] Finan B Capozzi ME Campbell JE. Repositioning glucagon action in the physiology and pharmacology of diabetes. Diabetes. (2019) 69:532–41. doi: 10.2337/dbi19-0004 PMC7085250 31178432

[16] Forbes JM Cooper ME. Mechanisms of diabetic complications. Physiol Rev. (2013) 93:137–88. doi: 10.1152/physrev.00045.2011 23303908

[17] Zhang J Liu F. Tissue-specific insulin signaling in the regulation of metabolism and aging. IUBMB Life. (2014) 66:485–95. doi: 10.1002/iub.v66.7 PMC4140976 25087968

[18] Ormazabal V Nair S Elfeky O Aguayo C Salomon C Zuñiga FA. Association between insulin resistance and the development of cardiovascular disease. Cardiovasc Diabetol. (2018) 17:122. doi: 10.1186/s12933-018-0762-4 30170598 PMC6119242

[19] Ruze R Liu T Zou X Song J Chen Y Xu R. Obesity and type 2 diabetes mellitus: connections in epidemiology, pathogenesis, and treatments. Front Endocrinol. (2023) 14:1161521. doi: 10.3389/fendo.2023.1161521 PMC10161731 37152942

[20] Hurtado MD Vella A. What is type 2 diabetes? Medicine. (2019) 47:10–5. doi: 10.1016/j.mpmed.2018.10.010

[21] Dludla PV Mabhida SE Ziqubu K Nkambule BB Mazibuko-Mbeje SE Hanser S. Pancreatic β-cell dysfunction in type 2 diabetes: Implications of inflammation and oxidative stress. World J Diabetes. (2023) 14:130–46. doi: 10.4239/wjd.v14.i3.130 PMC10075035 37035220

[22] Holt RI. Diagnosis, epidemiology and pathogenesis of diabetes mellitus: an update for psychiatrists. Br J Psychiatry. (2004) 184:s55–63. doi: 10.1192/bjp.184.47.s55 15056594

[23] DeFronzo RA Ferrannini E Alberti KGMM Zimmet P Alberti G. International textbook of diabetes mellitus Vol. 2. Hoboken, New Jersey: John Wiley & Sons (2015).

[24] Yan L. Pathogenesis of chronic hyperglycemia: from reductive stress to oxidative stress. J Diabetes Res. (2014) 2014:137919. doi: 10.1155/2014/137919 25019091 PMC4082845

[25] Poungvarin N Lee JK Yechoor VK Li MV Assavapokee T Suksaranjit P. Carbohydrate response element-binding protein (ChREBP) plays a pivotal role in beta cell glucotoxicity. Diabetologia. (2012) 55:1783–96. doi: 10.1007/s00125-012-2506-4 PMC4010252 22382520

[26] Germoush MO Elgebaly HA Hassan S Kamel EM Bin-Jumah M Mahmoud AM. Consumption of terpenoids-rich Padina pavonia extract attenuates hyperglycemia, insulin resistance and oxidative stress, and upregulates PPARγ in a rat model of type 2 diabetes. Antioxidants (Basel). (2019) 9:22. doi: 10.3390/antiox9010022 31887984 PMC7022299

[27] Newsholme P Cruzat VF Keane KN Carlessi R de Bittencourt PI Jr. Molecular mechanisms of ROS production and oxidative stress in diabetes. Biochem J. (2016) 473:4527–50. doi: 10.1042/BCJ20160503C 27941030

[28] Yan LJ. Redox imbalance stress in diabetes mellitus: Role of the polyol pathway. Anim Model Exp Med. (2018) 1:7–13. doi: 10.1002/ame2.2018.1.issue-1 PMC5975374 29863179

[29] Chen N Mu L Yang Z Du C Wu M Song S. Carbohydrate response element-binding protein regulates lipid metabolism via mTOR complex1 in diabetic nephropathy. J Cell Physiol. (2021) 236:625–40. doi: 10.1002/jcp.v236.1 32583421

[30] Nambirajan G Karunanidhi K Ganesan A Rajendran R Kandasamy R Elangovan A. Evaluation of antidiabetic activity of bud and flower of Avaram Senna (*Cassia auriculata* L.) In high fat diet and streptozotocin induced diabetic rats. BioMed Pharmacother. (2018) 108:1495–506. doi: 10.1016/j.biopha.2018.10.007 30372851

[31] Newsholme P Keane KN Carlessi R Cruzat V. Oxidative stress pathways in pancreatic β-cells and insulin-sensitive cells and tissues: importance to cell metabolism, function, and dysfunction. Am J Physiol Cell Physiol. (2019) 317:C420–C33. doi: 10.1152/ajpcell.00141.2019 31216193

[32] Asmat U Abad K Ismail K. Diabetes mellitus and oxidative stress-A concise review. Saudi Pharm J. (2016) 24:547–53. doi: 10.1016/j.jsps.2015.03.013 PMC5059829 27752226

[33] Akash MSH Rehman K Chen S. Role of inflammatory mechanisms in pathogenesis of type 2 diabetes mellitus. J Cell Biochem. (2013) 114:525–31. doi: 10.1002/jcb.v114.3 22991242

[34] Czech MP. Insulin action and resistance in obesity and type 2 diabetes. Nat Med. (2017) 23:804–14. doi: 10.1038/nm.4350 PMC6048953 28697184

[35] Samuel VT Shulman GI. The pathogenesis of insulin resistance: integrating signaling pathways and substrate flux. J Clin Investig. (2016) 126:12–22. doi: 10.1172/JCI77812 26727229 PMC4701542

[36] Zhao X An X Yang C Sun W Ji H Lian F. The crucial role and mechanism of insulin resistance in metabolic disease. Front Endocrinol. (2023) 14:1149239. doi: 10.3389/fendo.2023.1149239 PMC10086443 37056675

[37] Tan SY Mei Wong JL Sim YJ Wong SS Mohamed Elhassan SA Tan SH. Type 1 and 2 diabetes mellitus: A review on current treatment approach and gene therapy as potential intervention. Diabetes Metab Syndr. (2019) 13:364–72. doi: 10.1016/j.dsx.2018.10.008 30641727

[38] Gregg EW Sattar N Ali MK. The changing face of diabetes complications. Lancet Diabetes Endocrinol. (2016) 4:537–47. doi: 10.1016/S2213-8587(16)30010-9 27156051

[39] Ali MK Pearson-Stuttard J Selvin E Gregg EW. Interpreting global trends in type 2 diabetes complications and mortality. Diabetologia. (2022) 65:3–13. doi: 10.1007/s00125-021-05585-2 34837505 PMC8660730

[40] Demir S Nawroth PP Herzig S Ekim Üstünel B. Emerging targets in type 2 diabetes and diabetic complications. Adv Sci (Weinh). (2021) 8:2100275. doi: 10.1002/advs.202100275 34319011 PMC8456215

[41] Yau M Maclaren NK Sperling M. Etiology and pathogenesis of diabetes mellitus in children and adolescents. Endotext. (2018). MDText. com, Inc. Available online at: https://www.ncbi.nlm.nih.gov/books/NBK498653/ (accessed 9/6/2024).

[42] Bharti SK Krishnan S Kumar A Kumar A. Antidiabetic phytoconstituents and their mode of action on metabolic pathways. Ther Adv Endocrinol Metab. (2018) 9:81–100. doi: 10.1177/2042018818755019 29492244 PMC5813859

[43] Gong Q Zhang P Wang J Gregg EW Cheng YJ Li G. Efficacy of lifestyle intervention in adults with impaired glucose tolerance with and without impaired fasting plasma glucose: A *post hoc* analysis of Da Qing Diabetes Prevention Outcome Study. Diabetes Obes Metab. (2021) 23:2385–94. doi: 10.1111/dom.v23.10 PMC8429240 34212465

[44] Colberg SR Sigal RJ Yardley JE Riddell MC Dunstan DW Dempsey PC. Physical activity/exercise and diabetes: A position statement of the American diabetes association. Diabetes Care. (2016) 39:2065–79. doi: 10.2337/dc16-1728 PMC6908414 27926890

[45] Chaudhury A Duvoor C Reddy Dendi VS Kraleti S Chada A Ravilla R. Clinical review of antidiabetic drugs: implications for type 2 diabetes mellitus management. Front Endocrinol. (2017) 8:6. doi: 10.3389/fendo.2017.00006 PMC5256065 28167928

[46] Wherrett D Ho J Huot C. Diabetes Canada clinical practice guidelines expert committee: diabetes Canada 2018 clinical practice guidelines for the prevention and management of diabetes in Canada. Can J Diabetes. (2018) 42:S1–S325. Available online at: https://www.sciencedirect.com/journal/canadian-journal-of-diabetes/vol/42/suppl/S1 (accessed 9/6/2024).29650079

[47] Asif M. The prevention and control the type-2 diabetes by changing lifestyle and dietary pattern. J Educ Health Promot. (2014) 3:1. doi: 10.4103/2277-9531.127541 24741641 PMC3977406

[48] Doglikuu BD Abdulai A Yaseri M Shakibazadeh E Djazayery A Mirzaei K. Association of adherence to diabetics feeding recommendation with glycaemic control and with malnutrition risk among normal weight persons with type 2 diabetes in Ghana. Malays J Med Sci. (2021) 28:84–99. doi: 10.21315/mjms 33958963 PMC8075600

[49] Umpierre D Ribeiro PA Kramer CK Leitao CB Zucatti AT Azevedo MJ. Physical activity advice only or structured exercise training and association with HbA1c levels in type 2 diabetes: a systematic review and meta-analysis. Jama. (2011) 305:1790–9. doi: 10.1001/jama.2011.576 21540423

[50] American Diabetes Association. Standards of medical care in diabetes-2017 abridged for primary care providers. Clin Diabetes. (2017) 35:5–26. doi: 10.2337/cd16-0067 28144042 PMC5241768

[51] Romesh Khardori M. Type 2 diabetes mellitus treatment & management 2024. Available online at: https://emedicine.medscape.com/article/117853-treatment (accessed 9/6/2024).

[52] Elkhalifa AM Nazar M Ali SI Khursheed I Taifa S Ahmad Mir M. Novel therapeutic agents for management of diabetes mellitus: A hope for drug designing against diabetes mellitus. Life. (2024) 14:99. doi: 10.3390/life14010099 38255714 PMC10821096

[53] Silver B Ramaiya K Andrew SB Fredrick O Bajaj S Kalra S. ADSG guidelines: insulin therapy in diabetes. Diabetes Ther. (2018) 9:449–92. doi: 10.1007/s13300-018-0384-6 PMC6104264 29508275

[54] Dardano A Bianchi C Del Prato S Miccoli R. Insulin degludec/insulin aspart combination for the treatment of type 1 and type 2 diabetes. Vasc Health Risk Manage. (2014) 10:465. doi: 10.2147%2FVHRM.S40097 10.2147/VHRM.S40097 PMC4132254 25143741

[55] Susilawati E Levita J Susilawati Y Sumiwi SA. Review of the case reports on metformin, sulfonylurea, and thiazolidinedione therapies in type 2 diabetes mellitus patients. Med Sci (Basel). (2023) 11:50. doi: 10.3390/medsci11030050 37606429 PMC10443323

[56] Sola D Rossi L Schianca GP Maffioli P Bigliocca M Mella R. Sulfonylureas and their use in clinical practice. Arch Med Sci. (2015) 11:840–8. doi: 10.5114/aoms.2015.53304 PMC4548036 26322096

[57] Khunti K Chatterjee S Gerstein HC Zoungas S Davies MJ. Do sulphonylureas still have a place in clinical practice? Lancet Diabetes Endocrinol. (2018) 6:821–32. doi: 10.1016/s2213-8587(18)30025-1 29501322

[58] Kalra S Bahendeka S Sahay R Ghosh S Md F Orabi A. Consensus recommendations on sulfonylurea and sulfonylurea combinations in the management of type 2 diabetes mellitus - international task force. Indian J Endocrinol Metab. (2018) 22:132–57. doi: 10.4103/ijem.IJEM_556_17 PMC5838894 29535952

[59] Zaman MSZ Hassan PH Islam SMSI Ullah MAU Rabbani MGR. Comparative efficacy of insulin, biguanides and sulfonylureas in the glycemic control of newly diagnosed diabetes mellitus patients in Rajshahi, Bangladesh. J Biosci. (2023) 31:39–49. doi: 10.3329/jbs.v31i1.69533

[60] Tahrani AA Barnett AH Bailey CJ. Pharmacology and therapeutic implications of current drugs for type 2 diabetes mellitus. Nat Rev Endocrinol. (2016) 12:566–92. doi: 10.1038/nrendo.2016.86 27339889

[61] Keegan MT. Endocrine pharmacology. Pharmacol Physiol anesthesia: Elsevier;. (2019) p:708–31. doi: 10.1016/B978-0-323-48110-6.00036-3

[62] Fridlyand LE Jacobson DA Philipson LH. Ion channels and regulation of insulin secretion in human β-cells: a computational systems analysis. Islets. (2013) 5:1–15. doi: 10.4161/isl.24166 23624892 PMC3662377

[63] Derosa G Maffioli P. [amp]]alpha;-Glucosidase inhibitors and their use in clinical practice. Arch Med Sci. (2012) 8:899–906. doi: 10.5114/aoms.2012.31621 23185202 PMC3506243

[64] Hossain MA Pervin R. Chapter 34 - current antidiabetic drugs: review of their efficacy and safety. In: Bagchi D Nair S, editors. Nutritional and Therapeutic Interventions for Diabetes and Metabolic Syndrome, 2nd ed. Amsterdam, The Netherlands: Academic Press (2018). p. 455–73.

[65] Hedrington MS Davis SN. Considerations when using alpha-glucosidase inhibitors in the treatment of type 2 diabetes. Expert Opin Pharmacother. (2019) 20:2229–35. doi: 10.1080/14656566.2019.1672660 31593486

[66] Damkaci F Szymaniak AA Biasini JP Cotroneo R. Synthesis of thiazolidinedione compound library. Compounds. (2022) 2:182–90. doi: 10.3390/compounds2030013

[67] Lebovitz HE. Thiazolidinediones: the forgotten diabetes medications. Curr Diabetes Rep. (2019) 19:151. doi: 10.1007/s11892-019-1270-y PMC6881429 31776781

[68] Dubois M Vantyghem MC Schoonjans K Pattou F. Thiazolidinediones in type 2 diabetes. Role of peroxisome proliferator-activated receptor gamma (PPARgamma). Ann Endocrinol (Paris). (2002) 63:511–23. 12527853

[69] Rameshrad M Razavi BM Ferns GAA Hosseinzadeh H. Pharmacology of dipeptidyl peptidase-4 inhibitors and its use in the management of metabolic syndrome: a comprehensive review on drug repositioning. Daru. (2019) 27:341–60. doi: 10.1007/s40199-019-00238-7 PMC6593018 30674032

[70] Lee YS Jun HS. Anti-diabetic actions of glucagon-like peptide-1 on pancreatic beta-cells. Metabolism. (2014) 63:9–19. doi: 10.1016/j.metabol.2013.09.010 24140094

[71] Srinivasan BT Davies M. Glycaemic management of type 2 diabetes. Medicine. (2019) 47:32–9. doi: 10.1016/j.mpmed.2018.10.009

[72] Collins L Costello RA. Glucagon-Like Peptide-1 Receptor Agonists. [Updated 2024 Feb 29]. In: StatPearls [Internet]. Treasure Island (FL): StatPearls Publishing (2024). Available online at: https://www.ncbi.nlm.nih.gov/books/NBK551568/. (accesed 9/6/2024). 31855395

[73] Yao H Zhang A Li D Wu Y Wang CZ Wan JY. Comparative effectiveness of GLP-1 receptor agonists on glycaemic control, body weight, and lipid profile for type 2 diabetes: systematic review and network meta-analysis. Bmj. (2024) 384:e076410. doi: 10.1136/bmj-2023-076410 38286487 PMC10823535

[74] Simes BC MacGregor GG. Sodium-glucose cotransporter-2 (SGLT2) inhibitors: A clinician's guide. Diabetes Metab Syndr Obes. (2019) 12:2125–36. doi: 10.2147/DMSO.S212003 PMC6799898 31686884

[75] Hsia DS Grove O Cefalu WT. An update on sodium-glucose co-transporter-2 inhibitors for the treatment of diabetes mellitus. Curr Opin Endocrinol Diabetes Obes. (2017) 24:73–9. doi: 10.1097/MED.0000000000000311 PMC6028052 27898586

[76] Scheen AJ. SGLT2 inhibition: efficacy and safety in type 2 diabetes treatment. Expert Opin Drug Saf. (2015) 14:1879–904. doi: 10.1517/14740338.2015.1100167 26513131

[77] Gęgotek A Skrzydlewska E. Antioxidative and anti-inflammatory activity of ascorbic acid. Antioxidants (Basel). (2022) 11:1993. doi: 10.3390/antiox11101993 36290716 PMC9598715

[78] Mason SA Parker L van der Pligt P Wadley GD. Vitamin C supplementation for diabetes management: A comprehensive narrative review. Free Radic Biol Med. (2023) 194:255–83. doi: 10.1016/j.freeradbiomed.2022.12.003 36526243

[79] Asbaghi O Nazarian B Yousefi M Anjom-Shoae J Rasekhi H Sadeghi O. Effect of vitamin E intake on glycemic control and insulin resistance in diabetic patients: an updated systematic review and meta-analysis of randomized controlled trials. Nutr J. (2023) 22:10. doi: 10.1186/s12937-023-00840-1 36800965 PMC9936725

[80] Lerma E White WB Bakris G. Effectiveness of nonsteroidal mineralocorticoid receptor antagonists in patients with diabetic kidney disease. Postgrad Med. (2023) 135:224–33. doi: 10.1080/00325481.2022.2060598 35392754

[81] Picard C Poirier A Bélanger S Labonté A Auld D Poirier J. Proprotein convertase subtilisin/kexin type 9 (PCSK9) in Alzheimer's disease: A genetic and proteomic multi-cohort study. PloS One. (2019) 14:e0220254–e. doi: 10.1371/journal.pone.0220254 PMC6705826 31437157

[82] Banerjee Y Pantea Stoian A Cicero AFG Fogacci F Nikolic D Sachinidis A. Inclisiran: a small interfering RNA strategy targeting PCSK9 to treat hypercholesterolemia. Expert Opin Drug Saf. (2022) 21:9–20. doi: 10.1080/14740338.2022.1988568 34596005

[83] DeFronzo RA Triplitt CL Abdul-Ghani M Cersosimo E. Novel agents for the treatment of type 2 diabetes. Diabetes Spectr. (2014) 27:100–12. doi: 10.2337/diaspect.27.2.100 PMC4522879 26246766

[84] Gasbjerg LS Gabe MBN Hartmann B Christensen MB Knop FK Holst JJ. Glucose-dependent insulinotropic polypeptide (GIP) receptor antagonists as anti-diabetic agents. Peptides. (2018) 100:173–81. doi: 10.1016/j.peptides.2017.11.021 29412817

[85] Mathiesen DS Bagger JI Bergmann NC Lund A Christensen MB Vilsbøll T. The effects of dual GLP-1/GIP receptor agonism on glucagon secretion-A Review. Int J Mol Sci. (2019) 20:4092. doi: 10.3390/ijms20174092 31443356 PMC6747202

[86] Fisman EZ Tenenbaum A. The dual glucose-dependent insulinotropic polypeptide (GIP) and glucagon-like peptide-1 (GLP-1) receptor agonist tirzepatide: a novel cardiometabolic therapeutic prospect. Cardiovasc Diabetol. (2021) 20:225. doi: 10.1186/s12933-021-01412-5 34819089 PMC8613929

[87] Chavda VP Ajabiya J Teli D Bojarska J Apostolopoulos V. Tirzepatide, a new era of dual-targeted treatment for diabetes and obesity: A mini-review. Molecules. (2022) 27:4315. doi: 10.3390/molecules27134315 35807558 PMC9268041

[88] Scheen AJ. Dual GIP/GLP-1 receptor agonists: New advances for treating type-2 diabetes. Ann Endocrinol (Paris). (2023) 84:316–21. doi: 10.1016/j.ando.2022.12.423 36639119

[89] Fanshier AV Crews BK Garrett MC Johnson JL. Tirzepatide: A novel glucose-dependent insulinotropic polypeptide/glucagon-like peptide 1 receptor agonist for the treatment of type 2 Ddabetes: The first twincretin. Clin Diabetes. (2023) 41:367–77. doi: 10.2337/cd22-0060 PMC10338280 37456095

[90] Dereje B Nardos A. Dopamine 2 agonists for the management of type 2 diabetes: a systematic review and meta-analysis. J Diabetes Metab Disord. (2023) 22:931–43. doi: 10.1007/s40200-023-01230-4 PMC10638275 37975084

[91] Chamarthi B Cincotta AH. Effect of bromocriptine-QR therapy on glycemic control in subjects with type 2 diabetes mellitus whose dysglycemia is inadequately controlled on insulin. Postgrad Med. (2017) 129:446–55. doi: 10.1080/00325481.2017.1315290 28374645

[92] Ferrell JM Chiang JYL. Understanding bile acid signaling in diabetes: From pathophysiology to therapeutic targets. Diabetes Metab J. (2019) 43:257–72. doi: 10.4093/dmj.2019.0043 PMC6581552 31210034

[93] Sansome DJ Xie C Veedfald S Horowitz M Rayner CK Wu T. Mechanism of glucose-lowering by metformin in type 2 diabetes: Role of bile acids. Diabetes Obes Metab. (2020) 22:141–8. doi: 10.1111/dom.13869 31468642

[94] Sedgeman LR Beysen C Allen RM Ramirez Solano MA Turner SM Vickers KC. Intestinal bile acid sequestration improves glucose control by stimulating hepatic miR-182-5p in type 2 diabetes. Am J Physiol Gastrointest Liver Physiol. (2018) 315:G810–G23. doi: 10.1152/ajpgi.00238.2018 PMC6415711 30160993

[95] Laakso M Fernandes Silva L. Statins and risk of type 2 diabetes: mechanism and clinical implications. Front Endocrinol (Lausanne). (2023) 14:1239335. doi: 10.3389/fendo.2023.1239335 37795366 PMC10546337

[96] Eldor R Raz I. American Diabetes Association indications for statins in diabetes: is there evidence? Diabetes Care. (2009) 32 Suppl 2:S384–91. doi: 10.2337/dc09-S345 PMC2811452 19875586

[97] American Diabetes Association. 10. Cardiovascular disease and risk management: cardiovascular disease and risk management: *standards of medical care in diabetes*-2021. Diabetes Care. (2021) 44:S125–s50. doi: 10.2337/dc21-S010 33298421

[98] Adeghate E Kalász H. Amylin analogues in the treatment of diabetes mellitus: medicinal chemistry and structural basis of its function. Open Med Chem J. (2011) 5:78–81. doi: 10.2174/1874104501105010078 21966328 PMC3174573

[99] Akter R Cao P Noor H Ridgway Z Tu L-H Wang H. Islet amyloid polypeptide: structure, function, and pathophysiology. J Diabetes Res. (2016) 2016:2798269. doi: 10.1155/2016/2798269 26649319 PMC4662979

[100] Bahadoran Z Ghasemi A Mirmiran P Azizi F Hadaegh F. Beneficial effects of inorganic nitrate/nitrite in type 2 diabetes and its complications. Nutr Metab (Lond). (2015) 12:16. doi: 10.1186/s12986-015-0013-6 25991919 PMC4436104

[101] Fiorina P. GABAergic system in β-cells: from autoimmunity target to regeneration tool. Diabetes. (2013) 62:3674–6. doi: 10.2337/db13-1243 PMC3806604 24158998

[102] Jin Z Mendu SK Birnir B. GABA is an effective immunomodulatory molecule. Amino Acids. (2013) 45:87–94. doi: 10.1007/s00726-011-1193-7 22160261 PMC3680704

[103] Wan Y Wang Q Prud’homme GJ. GABAergic system in the endocrine pancreas: a new target for diabetes treatment. Diabetes Metab Syndr Obes. (2015) 8:79. doi: 10.2147/DMSO.S50642 25678807 PMC4322886

[104] Bhat R Axtell R Mitra A Miranda M Lock C Tsien RW. Inhibitory role for GABA in autoimmune inflammation. Proc Natl Acad Sci. (2010) 107:2580–5. doi: 10.1073/pnas.0915139107 PMC2823917 20133656

[105] Tegegne BA Kebede B. Probiotics, their prophylactic and therapeutic applications in human health development: A review of the literature. Heliyon. (2022) 8:e09725. doi: 10.1016/j.heliyon.2022.e09725 35785237 PMC9240980

[106] Sun Z Sun X Li J Li Z Hu Q Li L. Using probiotics for type 2 diabetes mellitus intervention: Advances, questions, and potential. Crit Rev Food Sci Nutr. (2020) 60:670–83. doi: 10.1080/10408398.2018.1547268 30632770

[107] Mishra SP Wang S Nagpal R Miller B Singh R Taraphder S. Probiotics and prebiotics for the amelioration of type 1 diabetes: Present and future perspectives. Microorganisms. (2019) 7:67. doi: 10.3390/microorganisms7030067 30832381 PMC6463158

[108] Li Y Liu T Qin L Wu L. Effects of probiotic administration on overweight or obese children: a meta-analysis and systematic review. J Transl Med. (2023) 21:525. doi: 10.1186/s12967-023-04319-9 37542325 PMC10401801

[109] Nagpal R Kumar A Kumar M Behare PV Jain S Yadav H. Probiotics, their health benefits and applications for developing healthier foods: a review. FEMS Microbiol Lett. (2012) 334:1–15. doi: 10.1111/j.1574-6968.2012.02593.x 22568660

[110] Gomes AC Bueno AA de Souza RGM Mota JF. Gut microbiota, probiotics and diabetes. Nutr J. (2014) 13:60. doi: 10.1186/1475-2891-13-60 24939063 PMC4078018

[111] AL-Moosawi Z Almahdawi M Al-Charak A. Camel milk as an integrated food and its physical and chemical properties with therapeutic characteristics. Int J Vet Sci Anim Husb. (2023) 8:90–6. doi: 10.22271/veterinary.2023.v8.i1b.474

[112] Wang X Zhang P Zhang X. Probiotics regulate gut microbiota: An effective method to improve immunity. Molecules. (2021) 26:6076. doi: 10.3390/molecules26196076 34641619 PMC8512487

[113] Kang Y Cai Y. The development of probiotics therapy to obesity: a therapy that has gained considerable momentum. Hormones (Athens). (2018) 17:141–51. doi: 10.1007/s42000-018-0003-y 29858841

[114] Van Poelje PD Potter SC Erion MD. Fructose-1, 6-bisphosphatase inhibitors for reducing excessive endogenous glucose production in type 2 diabetes. Handb Exp Pharmacol. (2011) 203:279–301. doi: 10.2337/db05-1443 21484576

[115] Kaur R Dahiya L Kumar M. Fructose-1,6-bisphosphatase inhibitors: A new valid approach for management of type 2 diabetes mellitus. Eur J Med Chem. (2017) 141:473–505. doi: 10.1016/j.ejmech.2017.09.029 29055870

[116] Srinivasan M Thangaraj SR Arzoun H. Gene therapy - can it cure type 1 diabetes? Cureus. (2021) 13:e20516. doi: 10.7759/cureus.20516 35004071 PMC8723777

[117] Wang Y Chen X Cao W Shi Y. Plasticity of mesenchymal stem cells in immunomodulation: Pathological and therapeutic implications. Nat Immunol. (2014) 15:1009–16. doi: 10.1038/ni.3002 25329189

[118] Cudini A Fierabracci A. Advances in immunotherapeutic approaches to type 1 diabetes. Int J Mol Sci. (2023) 24:9220. doi: 10.3390/ijms24119220 37298175 PMC10253167

[119] Qin L Liu N Bao C-l Yang D-z Ma G-x Yi W-h. Mesenchymal stem cells in fibrotic diseases-the two sides of the same coin. Acta Pharmacol Sin. (2023) 44:268–87. doi: 10.1038/s41401-022-00952-0 PMC9326421 35896695

[120] Farooq T Rehman K Hameed A Akash MSH. Stem cell therapy and type 1 diabetes mellitus: Treatment strategies and future perspectives. Adv Exp Med Biol. (2019) 1084:95–107. doi: 10.1007/5584_2018_195 29896720

[121] Du S Li Y Geng Z Zhang Q Buhler LH Gonelle-Gispert C. Engineering islets from stem cells: The optimal solution for the treatment of diabetes? Front Immunol. (2022) 13:869514. doi: 10.3389/fimmu.2022.869514 35572568 PMC9092457

[122] Ghoneim MA Gabr MM El-Halawani SM Refaie AF. Current status of stem cell therapy for type 1 diabetes: a critique and a prospective consideration. Stem Cell Res Ther. (2024) 15:23. doi: 10.1186/s13287-024-03636-0 38281991 PMC10823744

[123] Liang Y Hou D Zhao X Wang L Hu Y Liu J. Childhood obesity affects adult metabolic syndrome and diabetes. Endocrine. (2015) 50:87–92. doi: 10.1007/s12020-015-0560-7 25754912

[124] Xing R Li Q Xia L Song J Xu L Zhang J. Au-modified three-dimensional In_2_O_3_ inverse opals: synthesis and improved performance for acetone sensing toward diagnosis of diabetes. Nanoscale. (2015) 7:13051–60. doi: 10.1039/C5NR02709H 26172336

[125] Ma H Murphy C Loscher CE O'Kennedy R. Autoantibodies - enemies, and/or potential allies? Front Immunol. (2022) 13:953726. doi: 10.3389/fimmu.2022.953726 36341384 PMC9627499

[126] Ke Q Kroger CJ Clark M Tisch RM. Evolving antibody therapies for the treatment of type 1 diabetes. Front Immunol. (2020) 11:624568. doi: 10.3389/fimmu.2020.624568 33679717 PMC7930374

[127] Nagy G Szekely TE Somogyi A Herold M Herold Z. New therapeutic approaches for type 1 diabetes: Disease-modifying therapies. World J Diabetes. (2022) 13:835–50. doi: 10.4239/wjd.v13.i10.835 PMC9606789 36312000

[128] Kumar S Mittal A Babu D Mittal A. Herbal medicines for diabetes management and its secondary complications. Curr Diabetes Rev. (2021) 17:437–56. doi: 10.2174/18756417MTExfMTQ1z 33143632

[129] Patel DK Prasad SK Kumar R Hemalatha S. An overview on antidiabetic medicinal plants having insulin mimetic property. Asian Pac J Trop BioMed. (2012) 2:320–30. doi: 10.1016/S2221-1691(12)60032-X PMC3609288 23569923

[130] Tegegne BA Yihunie W Aschale Y Belew H Getachew M. Validation of blood glucose and lipid-lowering effect of solvent fractions of the *Crinum abyssinicum* shoot tips in streptozotocin-induced diabetic mice. Glob Adv Integr Med Health. (2024) 13:27536130231225464. doi: 10.1177/27536130231225464 38226326 PMC10788077

[131] Zhao Y Wu Y Wang M. Bioactive substances of plant origin. In: Cheung PCK, editor. Handbook of food chemistry. Springer Berlin Heidelberg, Berlin, Heidelberg (2015). p. 1–35.

[132] Kumar A Nirmal P Kumar M Jose A Tomer V Oz E. Major phytochemicals: Recent advances in health benefits and extraction method. Molecules. (2023) 28:887. doi: 10.3390/molecules28020887 36677944 PMC9862941

[133] Mabena P Fasemore TMD Nkomozepi P. Impact of nutraceuticals on type 1 and type 2 diabetes mellitus-induced micro- and macrovasculopathies. Appl Sci. (2024) 14:64. doi: 10.3390/app14010064

[134] Tran N Pham B Le L. Bioactive compounds in anti-diabetic plants: from herbal medicine to modern drug discovery. Biol (Basel). (2020) 9:252. doi: 10.3390/biology9090252 PMC7563488 32872226

[135] Bindu J Narendhirakannan RT. Role of medicinal plants in the management of diabetes mellitus: a review. 3 Biotech. (2019) 9:4. doi: 10.1007/s13205-018-1528-0 PMC6291410 30555770

[136] Saad B Kmail A Haq SZH. Anti-diabesity middle eastern medicinal plants and their action mechanisms. Evid Based Complement Alternat Med. (2022) 2022:2276094. doi: 10.1155/2022/2276094 35899227 PMC9313926

[137] Governa P Baini G Borgonetti V Cettolin G Giachetti D Magnano AR. Phytotherapy in the management of diabetes: A review. Molecules. (2018) 23:105. doi: 10.3390/molecules23010105 29300317 PMC6017385

[138] Modak M Dixit P Londhe J Ghaskadbi S Devasagayam TP. Indian herbs and herbal drugs used for the treatment of diabetes. J Clin Biochem Nutr. (2007) 40:163–73. doi: 10.3164/jcbn.40.163 PMC2275761 18398493

[139] Przeor M. Some common medicinal plants with antidiabetic activity, known and available in Europe (A mini-review). Pharm (Basel Switzerland). (2022) 15:65. doi: 10.3390/ph15010065 PMC8778315 35056122

[140] Shane-McWhorter L. Dietary supplements for diabetes: an evaluation of commonly used products. Diabetes Spectr. (2009) 22:206–13. doi: 10.2337/diaspect.22.4.206

[141] Hannon BA Fairfield WD Adams B Kyle T Crow M Thomas DM. Use and abuse of dietary supplements in persons with diabetes. Nutr Diabetes. (2020) 10:14. doi: 10.1038/s41387-020-0117-6 32341338 PMC7186221

[142] Chen W Balan P Popovich DG. Review of ginseng anti-diabetic studies. Molecules. (2019) 24:4501. doi: 10.3390/molecules24244501 31835292 PMC6943541

